# Integrating multi-layer perceptron and random forest in an ensemble framework for improved genomic prediction accuracy and SHAP-derived interpretability of residual feed intake in cattle

**DOI:** 10.1186/s40104-026-01476-x

**Published:** 2026-08-08

**Authors:** Edwin Jun Kiat Ong, Mark H. Mooney, Faisal I. Rezwan, Hui Wang, Masoud Shirali

**Affiliations:** 1 Institute for Global Food Security, School of Biological Sciences, Queen’s University Belfast, Belfast, UK; 2 Agri-Food and Biosciences Institute, Hillsborough, UK; 3 Department of Computer Science, Aberystwyth University, Aberystwyth, UK; 4 School of Electronics, Electrical Engineering and Computer Science, Queen’s University Belfast, Belfast, UK

**Keywords:** Ensemble, Feed efficiency, Genomic prediction, Machine learning, Multi-Layer Perceptron, Random Forest, SHapley Additive exPlanations

## Abstract

**Background:**

Feed efficiency (FE) is recognized as a vital component of sustainable dairy production, with residual feed intake (RFI) serving as a key metabolic indicator of FE independent of production levels. However, the genetic improvement of this complex trait is limited by the inability of conventional genomic Best Linear Unbiased Prediction (gBLUP) model to capture complex, non-linear genetic architectures and epistatic interactions. To address these limitations, this study aims to compare the predictive performance of machine learning (ML) approaches, specifically Random Forest (RF) and Multi-Layer Perceptron (MLP) models, against standard gBLUP using genomic data from 220 UK Holstein cows genotyped with the BovineSNP50 v3 BeadChip with 47,446 quality-controlled single nucleotide polymorphisms (SNPs), phenotyped for RFI from 1996–2023. SHapley Additive exPlanations (SHAP) were applied to interpret SNP feature importance from the ML models, and an ensemble framework was implemented to leverage the complementary strengths of RF and MLP.

**Results:**

While the gBLUP model exhibited moderate predictive performance, the RF model demonstrated greater stability and accuracy compared to gBLUP, and the MLP showed higher variance across random states. The ensemble framework achieved the highest coefficient of determination (*R*^2^ = 0.39) and lowest root mean squared error (RMSE = 0.086). SHAP interpretability analysis revealed distinct genomic architectures between the models. The RF model prioritized genes regulating mitochondrial oxidation and immune surveillance, including *SKINT1* and *PPARGC1A*, whereas the MLP model identified genes involved in lipid metabolism and fat storage, including *APCDD1* and *ADIPOR1*. A convergence of biological signals was observed between the models, with nine common SNPs and 32 consensus genes identified, including *ACOD1*,* CNTNAP2*, and core spliceosomal snRNAs.

**Conclusions:**

These results suggest that the genetic architecture of RFI may be explored by ML methods as they offer flexibility in mapping non-additive genetic effects. The reliability of genomic predictions for complex traits was enhanced when complementary computational strategies were leveraged in an ensemble framework, providing a focused set of candidate genes for future experimental validation. Whilst exploratory gene networks require validation in larger cohorts, the ensemble ML framework presented here offers an interpretable approach for dissecting the genetic architecture of complex production traits in livestock.

**Supplementary Information:**

The online version contains supplementary material available at 10.1186/s40104-026-01476-x.

## Background

Feed efficiency (FE) plays an essential role in modern dairy production, significantly impacting both the economic viability and environmental sustainability of the food system. Defined as the efficiency of an animal to convert feed inputs into desired outputs in milk and milk solids while maintaining health and reproductive capabilities, FE is determined by a wide variety of influences ranging from abiotic factors such as the amount or type of feed consumed and biotic factors such as parity, weight, as well as genetics. Sustainable dairy production can be achieved through enhancing FE by reducing resource usage, such as land use requirements and limiting greenhouse gas emissions per unit of produced milk [1]. While FE can be increased via methods such as precision agriculture management [2], improvements in genetics provide for a more long-term and sustainable solution. Residual feed intake (RFI) is frequently utilized as a primary metric of FE in dairy cattle as it serves as a key indicator of metabolic efficiency. RFI is the difference between an animal's actual and expected feed intake and is quantified after adjusting for factors such as milk yield, metabolic body weight, and body weight change [3]. As a metric, RFI is a suitable measure of FE, being independent of production level per unit of body weight. Furthermore, RFI has been reported as a moderately heritable trait, with estimates ranging between 0.130 and 0.400, rendering it suitable for genomic improvement [4, 5]. However, the genetic improvement of FE in dairy cattle has been shown to be challenging due to the complex, multifactorial nature of the trait, which is influenced by numerous physiological processes including digestion, metabolism, and nutrient partitioning [6]. To model these biological complexities associated with complex traits, robust computational frameworks are required to estimate breeding values accurately. Conventional genomic Best Linear Unbiased Prediction (gBLUP) models the relationship between individuals using the genetic relationship matrix (GRM) to represent kinship between individuals in a population and the trait. This provides an estimated breeding value (EBV) of the individual based on shared kinship and can be used to estimate the heritability of a trait based on the additive genetic variance of the trait [6, 7]. However, gBLUP assumes linearity between markers, and while epistatic interactions between loci can contribute to additive genetic variance accounted for by the GRM [7], the strictly non-additive components of epistasis are excluded from this framework. As a result, gBLUP overlooks instances where the combined interaction of multiple genes governing intricate biological processes has an aggregate impact on feed conversion that differs from the simple sum of their individual additive effects [8].

To circumvent such limitations regarding the capture of non-linear and epistatic interactions, machine learning (ML) methods have emerged as promising alternatives or complementary approaches to traditional statistical methods of genomic prediction and association such as gBLUP. The implementation of ML in genomics allows for a re-evaluation of complex traits such as RFI, incorporating both additive and epistatic genetic variance. For instance, the Random Forest (RF) approach can capture epistatic effects between single nucleotide polymorphisms (SNPs) relating to RFI by identifying descendant pairs where pairs of SNPs appear together more frequently in decision paths within trees [9]. Classical ML algorithms, including eXtreme Gradient Boosting, Light Gradient Boosting Machine, RF and deep learning have been successfully applied to construct predictive models using genotypic data to explain the phenotypic variance in production traits of dairy cattle [10]. By comparing these methods with the conventional gBLUP approach, their utility in capturing non-linear relationships and interactions among genetic markers has been shown, potentially revealing genetic associations that might otherwise be missed [11, 12]. While the application of ML to identify genomic regions associated with FE in dairy cattle appears promising, studies demonstrating the application of deep learning methods remain scarce [13]. Therefore, this study has compared, using genomic data from a dairy cattle cohort, the predictive performance of a conventional gBLUP model against three ML approaches: RF, a Multi-Layer Perceptron (MLP), a deep learning method, and an ensemble framework. By contrasting SNP associations and analysing flanking genes and biological pathways, this evaluation aims not only to provide a better predictive approach to the identification of more FE animals, but also to elucidate the fundamental metabolic mechanisms and drivers underlying FE in dairy cattle.

## Methods

### Animal phenotypic data

Data were obtained from the Northern Ireland Farm Animal Biobank (NIFAB) at the Agri-Food and Biosciences Institute (AFBI), Hillsborough, Northern Ireland, United Kingdom. All experiments and associated procedures were in compliance with the UK Animals (Scientific Procedures) Act 1986, Animal Research Reporting of In Vivo Experiments (ARRIVE) guidelines, and were approved by the AFBI Hillsborough Ethical Review Group. Animals included in the analysis were lactating Holstein cattle from various studies from 1996 to 2023 with an average of 88 d in milk post calving. There were 220 primiparous and multiparous dairy cattle that were housed indoors and fed a combination of forage and concentrate while milk data from each individual cattle were analysed to obtain the yield and its nutritional content. To account for the difference in conditions over this period, the estimation of RFI was conducted using a linear mixed effect model regression framework:

$${Y}_{ij}=\left({\beta}_{0}+ \sum_{k=1}^{n}{\beta}_{k}{X}_{k,ij}\right)+{u}_{i}+{\epsilon}_{ij}$$

${Y}_{ij}$ represents the $j^{\mathrm{th}}$ observed weekly total dry matter intake (DMI) of the $i^{\mathrm{th}}$ animal, ${\beta}_{0}$ the intercept and *n* represents the total fixed effects year, season, study, treatment, lactation number, week post gestation, week post calving, days in milk (DIM), metabolic body weight measured weekly, average weekly weight change, and net energy (NE). The individual identifier for the cattle (${u}_{i}$) was included as a random intercept to explicitly account for repeated, longitudinal measures within the same animal and to capture inter-individual variability. Consequently, this random effect which represents the overall deviation of an individual's actual feed intake from its expected intake across all repeated observations was extracted and utilised as the final, singular RFI phenotype per animal [9]. The significance of individual fixed effects within this model is summarised in Table S1, notably, most covariates reached statistical significance. The RFI values used in subsequent genomic analyses therefore represent the individual metabolic efficiency independent of production level, body size, and management conditions.

### Genomic data collection and quality control

Genomic data were obtained from the Holstein cattle using the BovineSNP50 v3 BeadChip (Illumina Inc., San Diego, CA, USA) consisting of 53,218 SNPs distributed across the genome. The SNPs obtained from the raw data were processed using PLINK 2.0 [14] and missing genotypes were subsequently imputed using Beagle software v.5.5 [15]. Leveraging a reference panel derived from our own population of 1,361 Holstein cattle genotyped using the BovineSNP50 v3 BeadChip, the missing genotypes of the 220 individuals were imputed. The genotypes underwent further quality control (QC) procedure, removing SNPs with a minor allele frequency below 0.01, a maximum per-variant missing genotype rate of 0.05 and variants deviating significantly from Hardy–Weinberg Equilibrium (HWE) *P*-value lower than 10^–6^. After quality control filtering, a total of 54,674 SNPs were reduced to 47,446 SNPs for subsequent analysis.

### Genomic prediction approaches

#### Model evaluation strategy

To ensure the robustness and stability of the genomic predictions, a consistent evaluation framework was implemented across all models. First, the dataset was partitioned into an 80% training set and a 20% independent out of sample test set. To assess model performance and minimize overfitting, a five-fold cross-validation strategy was applied. Furthermore, to account for potential variability and ensure the reliability of the results, this cross-validation process was repeated multiple times utilizing 10 distinct random states. Finally, the predictive performance of each final model was assessed on the out of sample test set, with model accuracy and error quantified using the Pearson correlation coefficient (*r*), coefficient of determination (*R*^2^), root mean squared error (RMSE), and mean absolute error (MAE). Analyses were performed on a high-memory Dell PowerEdge R6525 compute node (2 TiB RAM, dual AMD EPYC 7702 64-Core processors). Of the approaches evaluated, gBLUP was the least computationally demanding, while the exhaustive hyperparameter search strategies employed for the RF and MLP models were considerably more intensive, with RF incurring the greatest computational cost owing to the large grid search space explored. To quantify the contribution of each input feature (SNP) to the model's predictions, SHapley Additive exPlanations (SHAP) analysis was conducted using the shap package (v0.48.0) [16]. For the RF model, TreeExplainer was used, which computes the SHAP values by exploiting the tree structure of the ensemble. For the MLP model, DeepExplainer was employed, which provides efficient approximate SHAP values by decomposing predictions through the neural network layers. SHAP was not applied to the gBLUP model as the SNP effects estimated via back solving the GRM are linear allele substitution effects that are directly interpretable as marker importance scores, conducting SHAP analysis on gBLUP would be redundant. The mean SHAP value and the mean absolute values were evaluated to understand the influence of the SNP on RFI. To assess the significance of each SNP, the mean SHAP values, values across all samples were standardised into z-scores by subtracting the mean and dividing by the standard deviation of the SHAP value distribution across all SNPs, following the approach of Kotlarz et al. [13]. Two-tailed *P*-values were then derived from these z-scores by reference to the standard normal distribution. Furthermore, to account for multiple testing, derived *P*-values were corrected using the Benjamini–Hochberg False Discovery Rate (FDR) method. SNPs with an FDR-corrected *P*-value below the threshold of 0.05 were considered statistically significant.

#### Linear model

To obtain the genomic EBV representing RFI and to account for population structure, a gBLUP model was utilized using the Genome-wide Complex Trait Analysis tool (GCTA v1.94.1) [17]. A genomic based GRM was calculated from the QC-filtered SNPs to capture the total additive genetic relationship between all the individuals. The GRM was then fitted into a gBLUP model to estimate the EBV of all the animals using GCTA. In each fold, a gBLUP model was fitted to the training set to estimate the EBVs, the EBVs were then back-solved to estimate SNP effects. The EBVs for the out-of-sample testing set were then predicted by summing the SNP effects using PLINK 2.0 and compared against its EBV when calculated using the full GRM to assess the performance of the gBLUP model in predicting the EBV values of the test set.

#### Random Forest algorithm

In the present study RF analysis was conducted to predict the RFI EBV of the individual using SNPs. The EBV was generated from the Mixed Linear Model (MLM) using a GRM to correct for population structure for RFI observed in the dairy cattle. The computational framework for the RF regression model was developed using Python (version 3.10.5) [18] with scikit-learn (version 1.4.2) [19] packages to develop, optimize, and evaluate the regression model. In addition to the standard cross-validation scoring, the out-of-bag (OOB) prediction error for each model configuration was calculated. A grid of hyperparameters was defined to explore a wide range of model configurations, for Ntree (number of decision trees) were 100, 200, and 500. The Mtry parameters (maximum number of features at each node) were set at $\sqrt{p}$, 0.01p, 0.1p, 0.3p, and p, where p denotes the total number of SNPs available to the model (*p* = 47,446). The max tree depth was set to None, 5, 10, and 20, minimum samples required to split at an internal node were 2, 5, 10, and 20, minimum number of samples at a leaf node were 1, 3, 5, and 10, the splitting criteria used was squared error and various levels of pruning via minimum impurity decrease were set at 0, 0.01 and 0.1. The optimal number for Mtry and Ntree value was determined by evaluating the lowest OOB prediction error that stabilize for the RF model. These parameters were chosen to control the complexity of individual learners within the ensemble, by exploring shallow to deep trees, this approach can capture the complexity of the data especially when the sample size was small. The models were scored using negative mean squared error to optimize the model selection across all cross validation folds. The RF model was trained following the model evaluation strategy.

#### Artificial neural network

A MLP neural network was developed for regression analysis utilizing Keras (v.3.8.0) [20] with a TensorFlow (v2.4.1) [21] backend. Hyperparameter optimization was performed to identify the optimal model architecture and training parameters using KerasTuner (v.1.4.7) [22] hyperband algorithm. Lasso regression (L1) and Ridge regression (L2) regularization penalties, weight penalties applied to discourage model over-complexity, were tuned with values for both penalties searched logarithmically in the range of 1 × 10^−6^ to 1 × 10^−2^. For hidden layers, a selection of activation functions, specifically Rectified Linear Unit, Hyperbolic Tangent, and Exponential Linear Unit was evaluated. The number of units in the first dense layer, was optimized within a range of 32 to 512, incrementing in steps of 32. The dropout rate which manages the proportion of neurons randomly deactivated during each training step as a regularisation technique to reduce overfitting for the first layer and for subsequent layers was tuned from 0.0 to 0.5 in increments of 0.1. The model was configured to include between 1 to 10 additional hidden layers. For each of these additional hidden layers, the number of units was optimized in the range of 16 to 256, with a step of 16. A single dense layer employing a linear activation function was designated as the output layer for the regression task. The choice of optimizer where the algorithm used to iteratively update network weights to minimise prediction error included Adam, or Stochastic Gradient Descent, which was configured with a momentum of 0.9. The learning rate was subject to tuning, explored logarithmically between 1 × 10^−5^ and 1 × 10^−2^. The hyperparameter search space was broad and flexible to discover the optimal model architecture and training configuration without making restrictive assumptions about the complex genetic architecture of RFI. The tuner was configured with several settings with the optimization objective being the minimization of MAE. A maximum of 100 epochs (one epoch being one complete pass through the entire training dataset) was set for each trial. The reduction factor for the Hyperband algorithm was 3, and the number of Hyperband iterations was set to 2. This hyperparameter search space was intentionally broad to allow the data to determine the optimal model hyperparameters for this genomic dataset without a priori assumptions. The final MLP model was then constructed using the optimal parameters identified from the hyperparameter search and evaluated following the model evaluation strategy.

#### Ensemble model implementation

To further enhance the predictive performance and leverage the complementary strengths of the individual ML approaches, an ensemble model was applied. This model extracts the hyperparameters from the best-performing individual runs from the RF and MLP models described previously and then evaluates them across 10 different evaluation random states. For each random state, out-of-sample predictions were generated independently by the RF and MLP models on the held-out test set. The ensemble prediction for each test sample was then computed as the mean of the RF and MLP predicted values. This averaging strategy was deliberately chosen over more complex meta-learner stacking approaches due to the small sample size (*n* = 220) and a learned second-stage combiner would risk overfitting. Whereas uniform averaging leverages the complementary inductive biases of the two models reduces variance via RF bootstrap aggregation and bias reduction via MLP continuous function approximation. The assumption underlying this approach is that prediction errors from the two models are partially uncorrelated, such that averaging yields a net reduction in overall prediction error. Wilcoxon signed-rank test was then conducted as post-hoc pairwise comparisons to identify which models differed from another using the performance metrics.

### Marker comparison and gene ontology analysis

SNPs for predicting FE were extracted from each model for comparison and were selected based on their SHAP value, providing insights into the genomic regions most strongly associated with FE. The thresholding for each model differed in the models used, and downstream analysis was conducted on the significant markers using gene ontology analysis. The comparison between the markers was conducted via overlaps between the genomic region of the significant markers from the RF and MLP models. These were compared against documented quantitative trait loci (QTLs) from the Cattle QTL database [23] associated with RFI. The SNPs identified from each model were determined to be overlapping when an SNP from one model resided within the 100 kb flanking region of another SNP from another model, the genes from the overlapped SNPs were identified.

To find genes associated with the SNPs, SNPs were first mapped to the closest known variants according to the reported rsIDs by interrogating 1 kb flanking genomic regions via the Ensembl REST API (version 15.9) for features linked to the variation and other chromosome nomenclature adjustments. Subsequently, genes residing within a 100 kb flanking region above and below the variant were retrieved using the Ensembl REST API. The genes associated with the SNPs between each model were deemed to be in consensus when the SNPs encode the same genes by different models. The 100 kb window size was adopted to balance the need to capture nearby *cis*-regulatory elements while minimizing the inclusion of unrelated, distant genes, that were consistent with our understanding of linkage disequilibrium in similar cattle population [12]. Proximal genes were mapped to Gene Ontology (GO) terms using the *Bos taurus* European Bioinformatics Institute Gene Ontology Annotation Database (2019–03–18 release) and the GO-basic OBO ontology knowledgebase [24]. Genes were used as input in STRING (v2.2.0) plug-in [25] to model protein–protein interactions (PPI), and the centrality of network nodes was examined using the Network Analyzer (v3.3.2) plug-in within Cytoscape (v3.10.3) software [26]. The highest degree of centrality was identified to represent genes with the highest number of connections within the biological network. Gene Ontology (GO) enrichment and clustering was performed using the Cytoscape ClueGO (v2.5.10) [27] plug-in to identify the Biological Processes, Cellular Components, Immune System Processes, Molecular Functions and Kyoto Encyclopedia of Genes and Genomes (KEGG) pathways associated with SNPs for RFI. Further correction to the *P*-value was conducted via Benjamini–Hochberg FDR method, applying a significance threshold of α=0.05 in ClueGO to account for multiple testing. A GO term was considered enriched when the adjusted *P*-value for the term was below the threshold. Functional groups were determined using a kappa score threshold of 0.4.

## Results

### Genomic prediction using gBLUP for residual feed intake

**Fig. 1:**
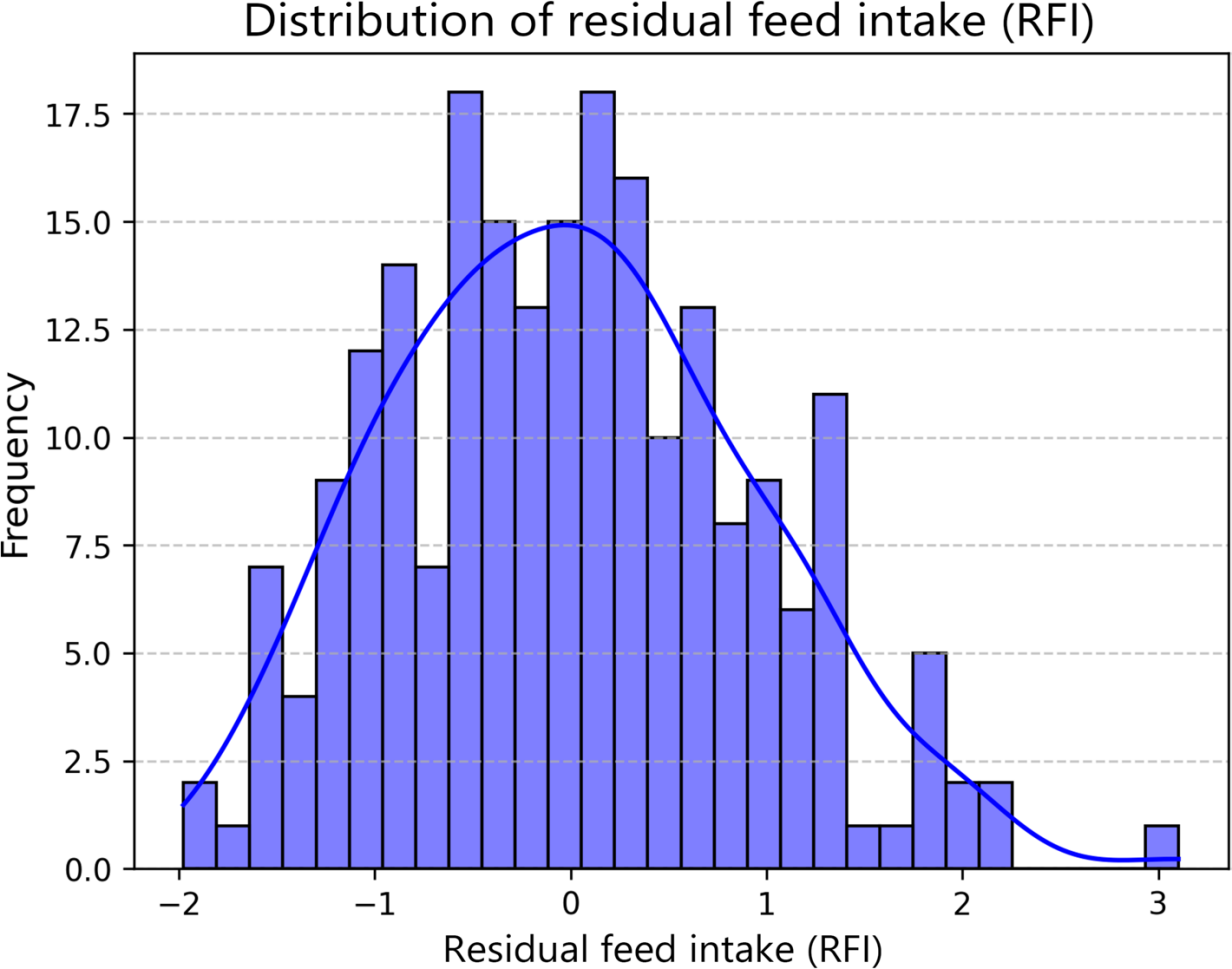
Distribution of residual feed intake (RFI) values of the frequency and variability across the entire herd including the individual with an RFI value of more than three standard deviations away from the mean

RFI values were determined using a linear mixed effect model from 220 cattle with their corresponding genotype. The RFI values were found to follow an approximately normal distribution with an interval of −1.7 to 2.0 with a single value removed that was considered an outlier with an RFI value of more than 3 standard deviation from the mean (Fig. 1). The negative RFI values represent cattle that have consumed less than expected feed intake for the same amount of milk production and highly positive values represent the excessive amount of feed consumed by the cattle. Therefore, high positive RFI values were regarded as inefficient cattle that did not effectively utilise feed resources whereas cattle with negative RFI values were determined to be feed-efficient individuals. After quality control filtering, a total of 54,674 SNPs were reduced to 47,446 SNPs for the subsequent linear model genome wide association with RFI. The variance component analysis using GCTA estimated the additive genetic variance (${\sigma}_{a}^{2}$) for RFI to be 0.079 ± 0.048, while the residual variance (${\sigma}_{e}^{2}$) was 0.75 ± 0.055. The resulting genomic heritability for RFI in this population was 0.095 ± 0.058. The predictive performance of the gBLUP model showed a moderate predictive performance values on the test set, yielding a mean *r* value of 0.63 ± 0.090, *R*^2^ of 0.23 ± 0.20, with prediction RMSE value of 0.094 ± 0.015 and a MAE value of 0.076 ± 0.013. To identify high-effect SNPs from the gBLUP model, marker effects were extracted and averaged across all cross-validation folds and random state repeats, and the top 100 SNPs ranked by absolute averaged effect magnitude were taken forward for comparison with ML-derived markers. The marker BTA-106641-no-rs, located on BTA 23 at position 36668732, had the largest effect size. No direct SNP-level overlap was observed between the top-ranked gBLUP SNPs and those prioritised by either the RF or MLP models.

### Random Forest hyperparameter selection and feature analysis

**Fig. 2:**
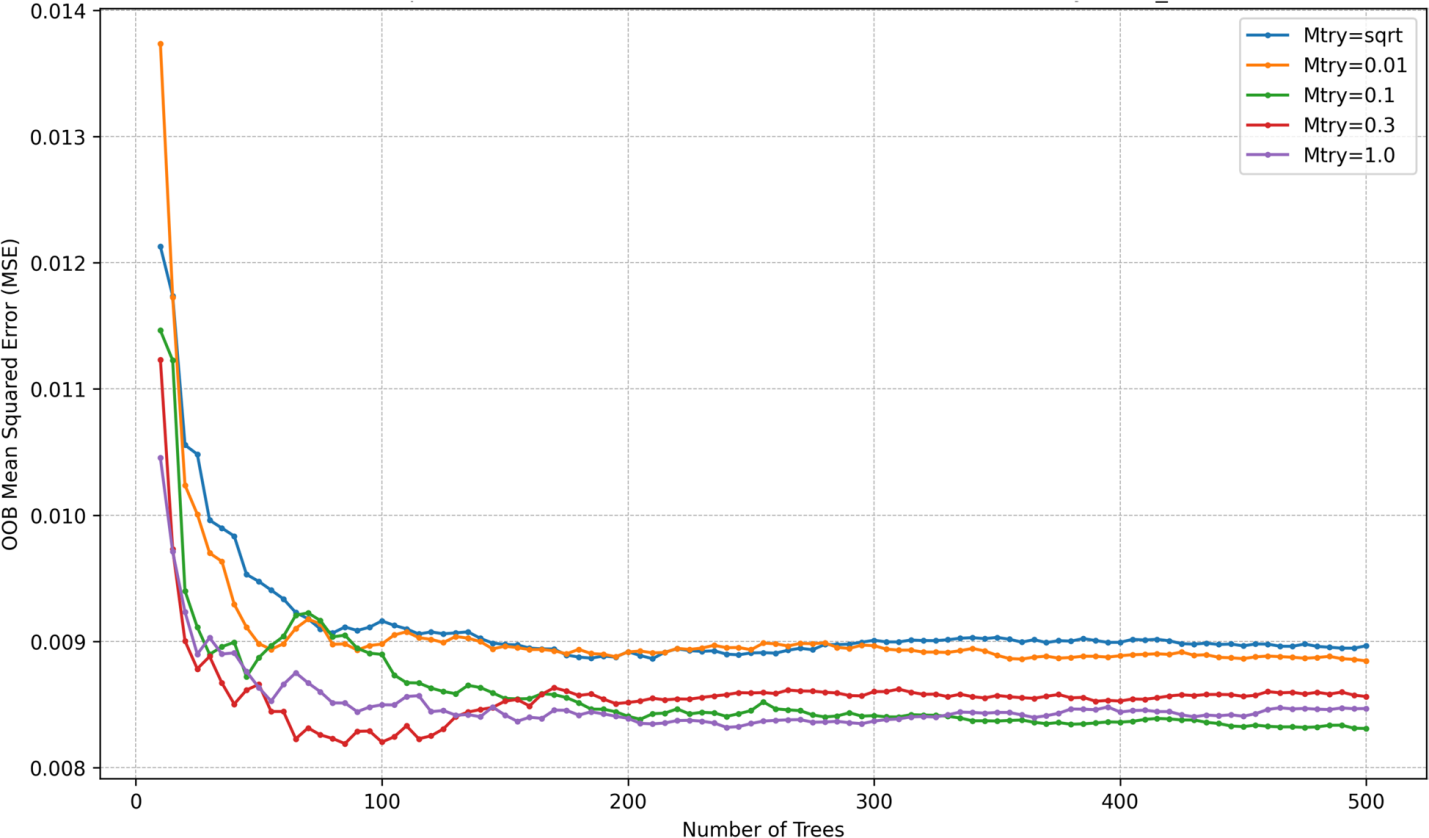
Out-of-bag (OOB) error rate plot for residual feed intake across various hyperparameters (number of SNPs randomly analysed per tree node (Mtry) and number of trees) for the Random Forest regression

The hyperparameters were selected using the OOB prediction error for the RF model, and the optimal number of trees was found to be around 500 trees where the OOB error stabilised (Fig. 2). The optimal number for Mtry which represents the number of SNPs randomly assessed per tree node was found to stabilise at 0.1*p* as it has the lowest OOB prediction error values followed by Mtry parameters of 0.01p, 0.3p and p while an Mtry of $\sqrt{p}$ had the worst predictive performance. Since an Mtry value of 0.1*p* provided the lowest OOB MSE score, it was selected as the hyperparameter of choice for the RF model and a Ntree value of 500 for the subsequent RF model. The final optimized model achieved an average *r* value of 0.73 ± 0.065, *R*^2^ of 0.35 ± 0.055, with prediction RMSE value of 0.088 ± 0.009 and a MAE value of 0.072 ± 0.008. Following the performance evaluation, the feature importance was determined using SHAP values from the optimized model to identify the most influential SNPs to the model prediction.

**Fig. 3:**
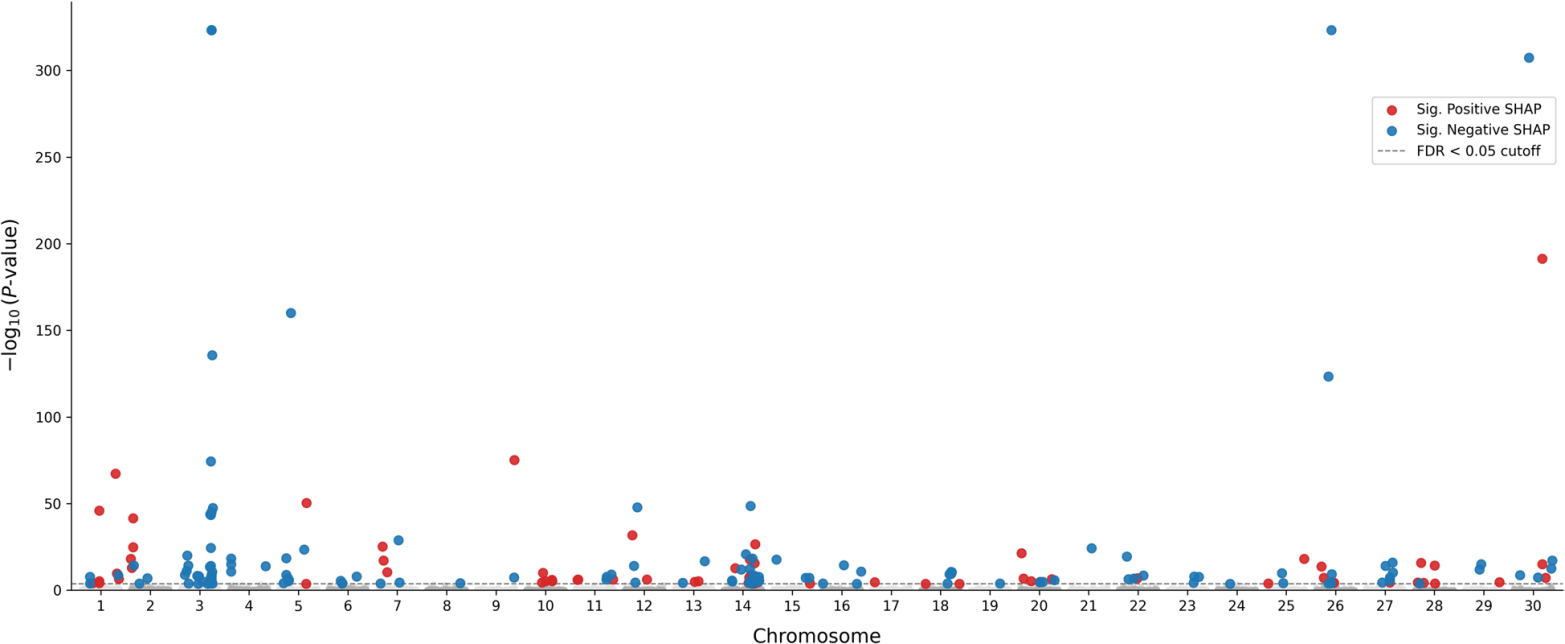
Manhattan plot with *P*-values generated by testing the SHAP values against the standard normal distribution for the best regression model using the Random Forest model. The red horizontal line represents the genome-wide significance threshold

**Fig. 4:**
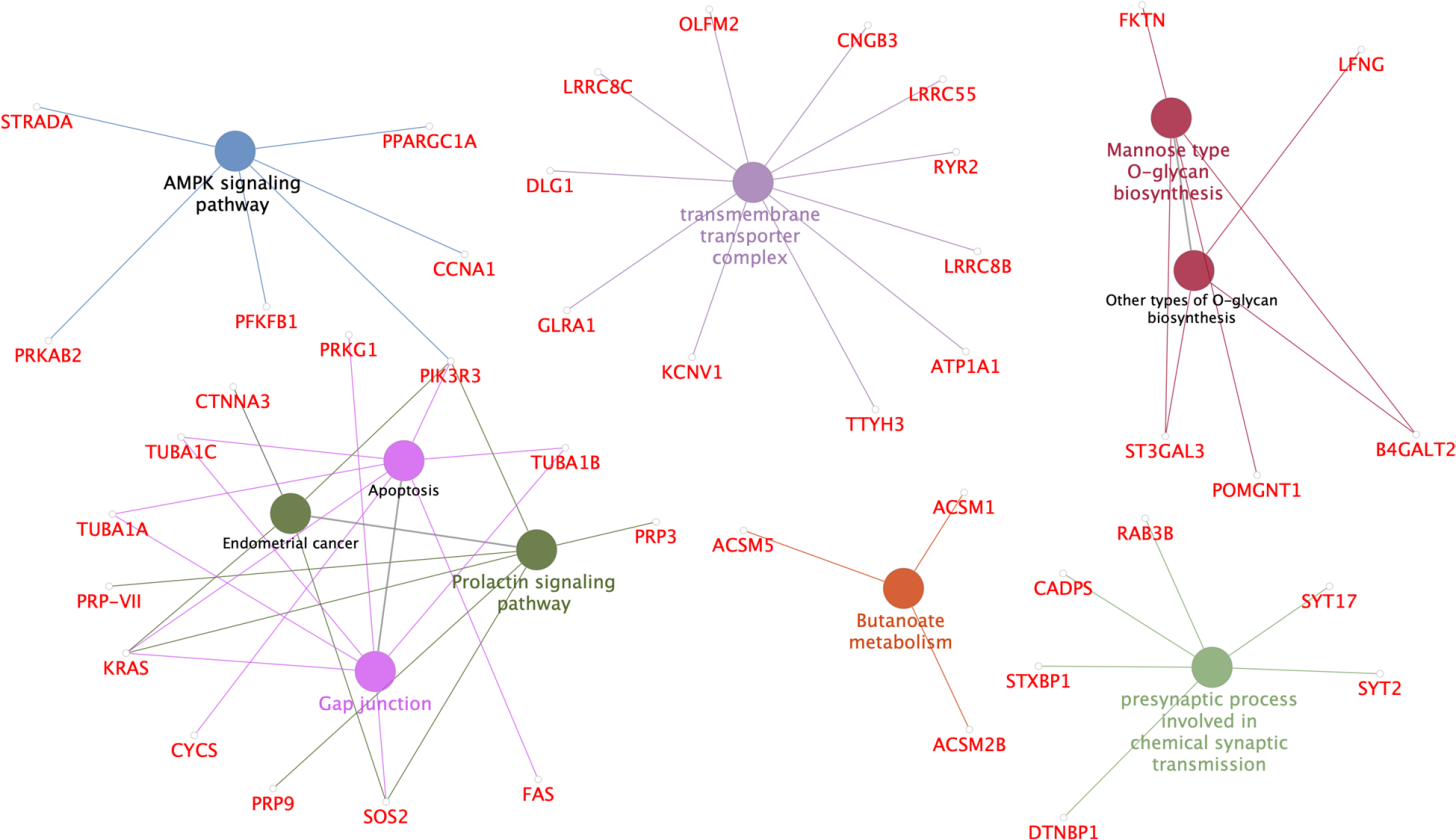
Enriched gene network for genes associated with residual feed intake in dairy cattle using a Random Forest model. Different node colours represent the functional groups in which the candidate genes are linked

Using the mean SHAP values to represent the feature importance, there were a total of 190 significant SNPs associated with RFI that were greater than the FDR-corrected *P*-value threshold of 0.05 (Fig. 3). These SNPs were located across all the chromosomes with some chromosomes having more markers as compared to other regions. The chromosome with the greatest number of SNPs found were in BTA 3 with 30 markers followed by BTA 14 with 24 markers found. The marker ARS-BFGL-NGS-30298 located in BTA 3 exhibited the highest absolute mean SHAP value (Fig. S1). This value was inversely associated with the trait, indicating that the SNP contributes to a decrease in RFI and characterizes more feed-efficient individuals. Although the *SKINT1* gene is located within a 100 kb window of this marker, the SNP itself represents a novel addition to SNP associated to RFI as it has not been previously documented in the Cattle QTL database. There were 190 SNPs associated with 373 genes and 23 SNPs were found within reported QTLs associated with RFI. From the input genes, STRING maps them on a PPI network, and the node with the highest degree of centrality was found to be the *FTSJ3* gene (Fig. S2). This gene was linked to the SNP BTB-00755453 located on BTA 19, other genes associated with this SNP included *CCDC47*,* DDX42*,* LIMD2*,* MAP3K3*,* PSMC5*,* SMARCD2*,* STRADA*,* TACO1* and *TCAM1*. The functional analysis revealed 10 significant pathways through GO enrichment, with 47 genes associated with these pathways. There was a varied number of GO terms, one GO Biological Processes terms (GO:0099531 ~ presynaptic process involved in chemical synaptic transmission), one GO Cellular Component (GO:0045178 ~ transmembrane transporter complex) and eight KEGG pathway (KEGG:00515 ~ Mannose type O-glycan biosynthesis, KEGG:00514 ~ Other types of O-glycan biosynthesis, KEGG:00650 ~ Butanoate metabolism, KEGG:04152 ~ AMPK signalling pathway, KEGG:04210 ~ Apoptosis, KEGG:04540 ~ Gap junction, KEGG:04917 ~ Prolactin signalling pathway and KEGG:05213 ~ Endometrial cancer) (Fig. 4).

### Multi-Layer Perceptron model

**Fig. 5:**
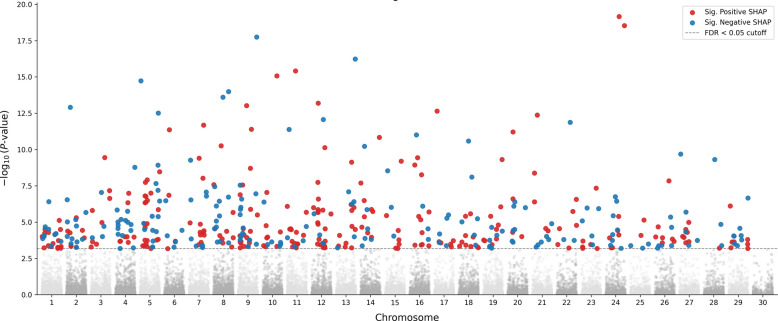
Manhattan plot with *P-*values generated by testing the SHAP values against the standard normal distribution for the best regression model using the Multi-Layer Perceptron model. The red horizontal line represents the genome-wide significance threshold

The Keras Tuner has explored up to 500 different model configurations in each run and the optimal hyperparameters for each random state was summarized (Table 1). The final optimized model was achieved with 4 hidden layers with the lowest out-of-sample test MSE value of an average *r* value of 0.66 ± 0.064, *R*^2^ of 0.33 ± 0.11, with prediction RMSE value of 0.089 ± 0.009 and a MAE value of 0.071 ± 0.007. From the 47,446 SNPs used in the best MLP model, 473 SNPs were significant, with a *P*-value below the FDR-corrected threshold of 0.05 (Fig. 5). The 473 of those SNPs could be annotated to a total of 947 genes by finding genes that were within 100 kb of its flanking regions. Located on BTA 24, marker ARS-BFGL-NGS-29353 showed the greatest absolute mean SHAP value (Fig. S3). The positive direction of this influence correlates with higher RFI, signalling a genetic link to reduced FE. Although APCDD1, NAPG, and PIEZO2 were identified within 100 kb of ARS-BFGL-NGS-29353, no corresponding entries were found in the Cattle QTL database, marking this as an additional novel locus for RFI. From the significant SNP, 69 SNPs were found within reported QTLs associated with RFI. After mapping the associated genes on a PPI network, the node with the highest degree of centrality was found to be the *TP53* gene followed by the *SRC* gene (Fig. S4). Using gene ontology enrichment, there were no other terms that were enriched due to the large number of SNPs captured.

**Table 1:** Top 3 most optimal Multi-Layer Perceptron architecture estimated for each random state

Random state	Test MSE	Test MAE	Test RMSE	Test *R*^2^	Training epoch	Optimizer	Learning rate
45	0.0056	0.061	0.075	0.51	100	Stochastic Gradient Descent	0.0018
46	0.0063	0.064	0.080	0.45	100	Stochastic Gradient Descent	0.0035
48	0.0058	0.062	0.076	0.49	100	Adam	0.000049

### Ensemble model and comparative analysis

**Fig. 6:**
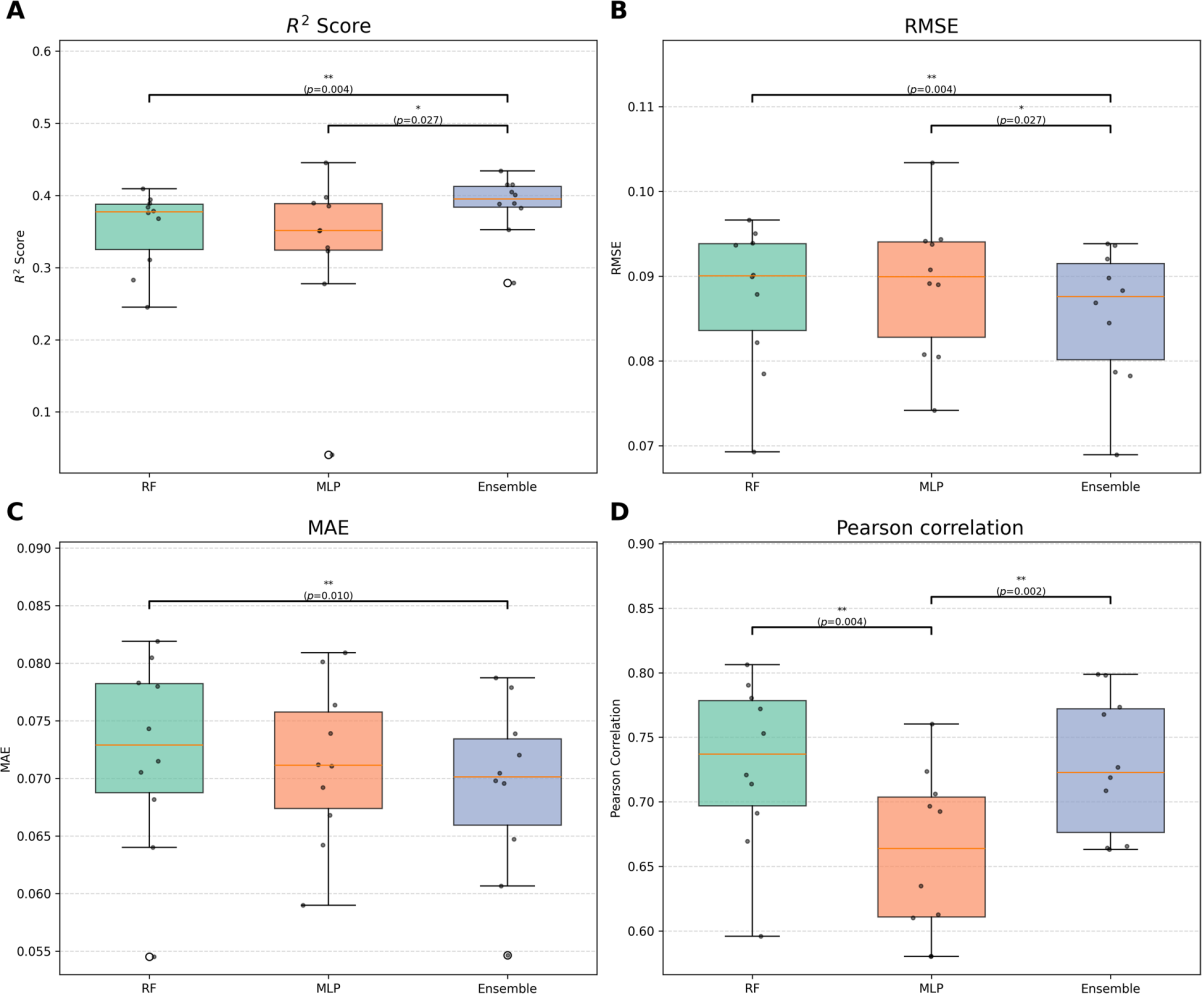
Comparison of model performance across 10 random different seeds. The box plots illustrate the distributions of (**A**) coefficient of determination (*R*^2^), **B** root mean squared error (RMSE), **C** mean absolute error (MAE), and **D** pearson correlation coefficient (*r*) for the Random Forest (RF), Multi-Layer Perceptron (MLP), and Ensemble models. Pairwise comparisons were conducted using the Wilcoxon signed-rank test with only statistically significant differences (*P* < 0.05) are annotated with brackets. Significance levels are denoted as: ^*^P < 0.05, ^**^P < 0.01, ^***^P < 0.001

Additionally, the predictive performance of the ensemble model by averaging predictions from RF and MLP was evaluated. The ensemble approach achieved a mean *r* value of 0.73 ± 0.054 and the highest *R*^2^ of 0.39 ± 0.044 (Fig. 6) with a lower error metrics of a RMSE of 0.086 ± 0.008 and a MAE value of 0.069 ± 0.008. There were several overlapping SNPs and genes between models with some SNPs located within reported QTL regions that were associated with RFI from the Cattle QTL database (Table 2). The MLP model identified the greatest number of SNPs overlapped between reported QTL regions. Furthermore, between the RF and MLP models, there were nine SNPs from each model that were in common, which were associated with 32 genes. These genes can be categorized by their biological functions which were *ACOD1* linked to the metabolic regulation of the immune system, the transcription processes such as the *U1*,* U2*,* U4* and *U6* snRNAs which encodes core components of the spliceosome, *PRKG1* gene associated with signalling and *CNTNAP2* gene that regulates appetite in cattle.

**Table 2:** Comparative summary of SNPs and genes identified by the Multi-Layer Perceptron (MLP) and Random Forest (RF) models against quantitative trait loci (QTLs) in the Cattle QTL database

Model	Total number of significant SNPs	Total number of genes	Candidate genes in QTL region	Overlapping SNPs between models^1^	Consensus genes from both models^2^
Multi-Layer Perceptron (MLP)	473	947	69 genes	Hapmap40228-BTA-88147 ARS-BFGL-NGS-114594 ARS-BFGL-NGS-1251 ARS-BFGL-BAC-15016 ARS-BFGL-NGS-36563 BTB-00640892 Hapmap53843-rs29014653 ARS-BFGL-NGS-39445	*ACOD1*,* AMMECR1L*,* B3GLCT*,* BICD1*,* CNNM1*,* CNTNAP2*,* DHH*,* EEA1*,* ELP2*,* IRGC*,* KCNN4*,* KCTD12*,* LMBR1L*,* LYPD5*,* PLEKHG7*,* POLR2D*,* PRKDC*,* PRKG1*,* RPRD1A*,* SLC39A6*,* SMG9*,* SNORA70*,* STAMBPL1*,* TUBA1A*,* TUBA1B*,* TUBA1C*,* U1*,* U2*,* U4*,* U6*,* WDR33*,* ZNF283*
Random Forest (RF)	190	373	23 genes	BTB-01739851 BovineHD0500006566 BovineHD0500008963 ARS-BFGL-NGS-53461 BTA-83050-no-rs BTB-00495415 ARS-BFGL-NGS-40844 ARS-BFGL-BAC-28016 ARS-BFGL-NGS-56400	

^1^Overlapping SNPs were identified when a marker from one model resided within a 100 kb flanking region of a marker from the other model

^2^Consensus genes are defined as genes associated with the overlapping SNPs that were shared between both models

## Discussion

### Modelling RFI using linear and machine learning models

In the evaluation of genomic prediction strategies for RFI, distinct variations in predictive capability were observed across applied methodologies highlighting the limitations of assuming strict additive effects in complex traits [28]. The conventional gBLUP model exhibited moderate predictive performance but was outperformed by ML approaches suggesting that the linear framework of gBLUP was unable to capture the complex epistatic interactions and non-linear relationship that characterizes the genetic architecture of metabolic efficiency [29, 30]. The RF model demonstrated greater stability and accuracy compared to gBLUP, with this advantage attributed to the RF algorithm’s recursive branching process, which naturally models epistasis. A split at a parent node creates a conditional dependency for subsequent child nodes, effectively mapping SNP-SNP interactions without requiring a priori specification [9]. Furthermore, the RF model’s reliance on bootstrap aggregation mitigated the impact of outliers and reduced variance, resulting in consistent performance metrics across random states. This consistency suggests that RF modelled the genetic architecture of RFI by prioritizing stable, high-impact loci rather than fitting noise [31, 32].

In contrast, the MLP model exhibited a stochastic performance profile characterized by higher variance across random states. Whilst achieving greater predictive accuracy in specific iterations, the MLP’s overall reliability was attenuated by susceptibility to overfitting. This behaviour is intrinsic to deep neural network architectures when applied to high-dimensional, limited-sample genomic datasets as their vast parameter space allows them to memorize noise alongside signal, leading to generalization challenges [33]. However, this high sensitivity led to a significantly broader spectrum of SNPs identified compared to the RF model, attributed to its capacity as a universal approximator [34]. Unlike decision trees which partition feature space, the MLP captures global non-linear dependencies and subtle low-effect variants through continuous activation functions [35]. Consequently, while less robust due to the limited number of samples, the MLP served as a potent heuristic tool for unravelling the complex architecture of RFI, detecting latent epistatic signals that are obscure to linear frameworks [36]. Finally, the ensemble model yielded the most optimal predictive performance, achieving the highest *R*^2^ value of 0.39 and the lowest RMSE of 0.086. Although the RF model achieved a marginally higher *r* value it was not significantly higher, and the ensemble approach thus provided the best explanatory power and lowest error. This improvement reinforces the concept of complementary inductive biases, while RF minimizes variance through averaging discrete decision trees, and MLP reduces bias through continuous function approximation, combining them effectively reduces their uncorrelated errors [37]. These findings indicate that the reliability of genomic predictions for complex traits like RFI can be significantly enhanced when complementary computational strategies are leveraged in an ensemble framework.

A key consideration for the translational application of these ML models in genomic selection pipelines is the sensitivity of the MLP architecture to dataset size and composition. The optimal number of hidden layers and neurons identified in this study is expected to shift as additional phenotypic and genotypic data are incorporated, a known challenge of deep learning applied to high-dimensional, low-sample genomic datasets [33]. To address this within the present framework, KerasTuner Hyperband was employed as an automated hyperparameter optimisation strategy, ensuring that model architecture is systematically re-evaluated rather than fixed to parameters derived from a single training configuration. In a prospective breeding pipeline, this re-optimisation step would be repeated as cohort size increases, allowing the model to adapt to the addition of data without manual reconfiguration. Regarding the interpretability of ML models, SHAP decompose each prediction into individual SNP contributions by exploiting the internal structure of each model, allowing the biological relevance of model outputs to be interrogated at the feature level [16]. For a stable genomic selection pipeline, SNP consistently prioritised across both model classes form the most robust feature set for downstream biological investigation. This consensus-based filtering approach partially decouples the final biological interpretation from the architectural instability inherent to the MLP, providing a more robust foundation for future experimental validation and integration into breeding programmes.

### Candidate genes and enriched pathways associated with RFI

The gene list identified from the significant markers established through the RF and MLP models reveals distinct genomic architectures underlying metabolic efficiency (Table S2). Genes regulating mitochondrial oxidation and energy expenditure were prioritized by the RF model. The marker ARS-BFGL-NGS-30298 on BTA 3 exhibited the highest absolute mean SHAP value and lies within the 100 kb flanking region of *SKINT1* (selection and upkeep of intraepithelial T cells 1), a gene involved in intraepithelial T-cell selection. [38]. This represents a novel locus for RFI not previously documented in Cattle QTL database and represents a compelling candidate for future validation. Other high ranking RF markers were proximal to genes associated with mitochondrial fatty acid oxidation (*PPARGC1A*, *CPT2*, *ACSM*). Furthermore, the *FTSJ3* gene located on BTA 19 had the highest-degree node in the RF PPI network (Fig. S2), it encodes a pre-rRNA processing protein implicated in ribosome biogenesis and translation regulation [39]. Enriched pathways included butanoate metabolism (KEGG:00650), consistent with butyrate's role as an energy substrate for rumen epithelial cells in efficient cattle [40], and the AMPK signalling pathway (KEGG:04152), a central regulator of cellular energy homeostasis [41].

In contrast, 473 significant SNPs were identified by the MLP model to capture complex, non-linear interactions, and linked to 947 genes involved in lipid synthesis, storage, and digestion (Table S3). A substantial proportion of these (69 SNPs) were located within previously reported QTLs for RFI, providing partial external validation. The highest-ranked marker, ARS-BFGL-NGS-29353 on BTA 24, was proximal to *APCDD1* gene, a potent inhibitor of the Wnt signalling pathway known to promote fat storage [42]. Additional candidate genes included *ADIPOR1* (adiponectin receptor 1) gene, a key receptor for adiponectin that mediates insulin-sensitizing effects in peripheral tissues [43] and, *PRKAG1* (AMPK subunit gamma-1) that was found to influence insulin sensitivity and promoting catabolism when ATP levels are low. Genes relating to insulin pathways have been known to be highly linked to the sensation of satiety due to insulin signalling in low RFI animals, indicating their importance as indicators of feed efficient individuals [42, 44, 45].Other SNPs were associated with genes *HMGCR* encoding the rate limiting enzyme in cholesterol biosynthesis, *AGPAT2* encoding an enzyme for lipid formation, and *PNLIP* that were linked to lipid metabolism [46–48]. Fat mobilization plays a crucial role in the ability to adapt to energy needs for milk production during the lactation period, thus genes linked to fat deposition and metabolism are pivotal in understanding FE in lactating cattle [49]. TP53 was the highest-degree node in the MLP PPI network, consistent with its known role in metabolic stress regulation [50–52].

### Convergent genetic signals and biological implications

In this study, a convergence of signals was observed between the RF and MLP models, with nine common SNPs identified that were associated with 32 consensus genes. The overlap between the algorithms trained on the same dataset provides some indication of consistent signal prioritisation across different modelling frameworks for predicting RFI [53, 54]. Among the consensus genes, aconitate decarboxylase 1 (*ACOD1*) is involved in the production of itaconate from the Krebs cycle during inflammation [55] and *CNTNAP2* has been linked to diet-induced obesity and energy expenditure in animal models [56]. Since RFI is defined as the difference between actual and expected feed intake, genetic variation in satiation signalling and appetite control could distinguish efficient from inefficient animals. A distinct group of consensus genes involved in transcriptional and post-transcriptional regulation was observed, specifically the small nuclear RNAs (snRNAs) *U1*,* U2*,* U4*, and *U6*, and the small nucleolar RNA *SNORA70*, all core components of the spliceosome and regulatory machinery. These genes have been previously associated with production traits and feed-related phenotypes in cattle [57–59], and their recurrence across both models may reflect a role for post-transcriptional regulation in metabolic efficiency [60–62]. Such post-transcriptional flexibility may allow efficient cattle to produce diverse protein isoforms that are better optimized for metabolic adaptation under varying physiological conditions [63].

The top-ranked SNP from the gBLUP model (BTA-106641-no-rs, BTA 23, position 36668732) was found to reside within QTL region QTL ID 5,303, previously identified for RFI in beef cattle by Sherman et al. [64] in a Canadian fine-mapping study using Angus, Charolais, and University of Alberta Hybrid cattle. While cross-species and crossbreed comparisons are inherently limited by differences in genotyping platforms, reference genome assemblies, and population structure, this chromosomal co-localisation provides supportive evidence for the biological relevance of this region. The top-ranked SNPs from the RF and MLP models were not reported among lead loci in previous RFI studies, suggesting they represent novel candidates in dairy cattle. However, the genomic regions the model tagged were functionally plausible as APCDD1-containing regions have been implicated in feed-efficiency-related QTL in beef populations, and PIEZO2, identified in proximity to the top MLP marker, encodes a mechanosensitive channel with established roles in homeostatic and feed intake-relevant pathways. It is also essential to recognise a sample size 220 animals may be considered limited, but the scale of this dataset reflects the constraints of working with longitudinal, phenotypically intensive RFI measurement. This sample size is consistent with those reported in previous RFI studies in dairy cattle; notably, Yao et al. [9] developed a Random Forest model for RFI prediction using 395 Holstein cows and 42,275 SNP genotypes. The convergence of signals across two independent ML algorithms nonetheless provides additional biological plausibility to the identified loci, and future validation in larger, independent cohorts will be critical before these targets can be integrated into genomic selection programs.

## Conclusion

This study indicates that ML approaches, specifically RF and MLP, represent promising tools to identify complex, non-linear genetic variants associated with RFI that remain elusive to conventional linear models such as gBLUP. While a larger dataset is required to validate the gene networks identified, presented findings highlight that integrating these distinct methodologies into an ensemble achieves the best predictive performance, suggesting the framework may better accommodate complex dependencies between genetic variants. Biologically, the RF model prioritized mitochondrial and translational regulators such as *SKINT1* and *FTSJ3*, emphasizing the role of the AMPK signalling and butanoate metabolism pathways in energy expenditure, while the MLP model identified key lipid metabolism and stress response genes such as *APCDD1* and *TP53* which facilitate efficient fat mobilization and adaptation. The identification of consensus markers within reported QTLs, alongside key genes such as the spliceosome components *U1*,* U2*,* U6* and the regulator *SNORA70*, highlights the biological signals that are resilient to model-specific artifacts. While this methodology has identified complex, non-additive genetic interactions governing FE, these initial gene networks are exploratory with further studies utilizing larger independent cohorts are required to validate these targets before full integration into genomic selection programs.

## Supplementary Information

Additional file 1: Table S1. Fixed effects of covariates on total dry matter intake (kg DM/Wk) from the linear mixed effects model used to derive residual feed intake (RFI). Table S2. Summary of 190 SNPs associated with residual feed intake (RFI) using the Random Forest model. Table S3. Summary of 473 SNPs associated with residual feed intake (RFI) using the Multi-Layer Perceptron model. Fig. S1. Feature importance of top-ranked SNPs ordered by their mean absolute SHapley Additive exPlanations (SHAP) values derived from the Random Forest model. Blue bars indicate markers associated with a decrease in the estimated breeding value (EBV) for residual feed intake (positive effect), while pink bars indicate markers associated with an increase in EBV (negative effect). Fig. S2. STRING interaction network for genes linked to SNPs associated using Random Forest to residual feed intake in dairy cattle with the highest degree of centrality represented by node *FTSJ3*. Fig. S3. Feature importance of top-ranked SNPs ordered by their mean absolute SHapley Additive exPlanations (SHAP) values derived from the Multi-Layer Perceptron model. Blue bars indicate markers associated with a decrease in the estimated breeding value (EBV) for residual feed intake (positive effect), while pink bars indicate markers associated with an increase in EBV (negative effect). Fig. S4. STRING interaction network for genes linked to SNPs associated using Multi-Layer Perceptron to residual feed intake in dairy cattle with the highest degree of centrality represented by node *TP53*.

## Data Availability

Data supporting this study are available on request from the Northern Ireland Farm Animal Biobank (NIFAB) lead Masoud Shirali. Access to data would be subject to approval after a data sharing agreement has been established.

## Abbreviations

EBV: Estimated breeding value
FDR: False Discovery Rate
FE: Feed efficiency
gBLUP: genomic Best Linear Unbiased Prediction
GO: Gene Ontology
GRM: Genomic relationship matrix
KEGG: Kyoto Encyclopedia of Genes and Genomes
MAE: Mean absolute error
ML: Machine learning
MLP: Multi-Layer Perceptron
MSE: Mean squared error
OOB: Out-of-bag
QTL: Quantitative trait locus
RF: Random Forest
RFI: Residual feed intake
RMSE: Root mean squared error
SHAP: SHapley Additive exPlanations
SNP: Single nucleotide polymorphism
