# Supplementary material for: Integrating multi-layer perceptron and random forest in an ensemble framework for improved genomic prediction accuracy and SHAP-derived interpretability of residual feed intake in cattle
Source: J Anim Sci Biotechnol. 2026 Aug 8;17:160. doi: 10.1186/s40104-026-01476-x (PMC13452126; doi:10.1186/s40104-026-01476-x)
Supplement: Supplementary file 1 [file 40104_2026_1476_MOESM1_ESM.docx]

Table S1: Fixed effects of covariates on total Dry Matter Intake (kg DM/Wk) from the linear mixed effects model used to derive Residual Feed Intake (RFI).

| Covariate | Estimate ($\beta$)^1^ | z-value | *P*-value^2^ | 95% CI Lower | 95% CI Upper |
| --- | --- | --- | --- | --- | --- |
| Season^3^ |  |  |  |  |  |
| Spring | 0.23 | 1.88 | 0.06 | -0.01 | 0.46 |
| Summer | 0.44 | 2.54 | 0.01* | 0.10 | 0.77 |
| Winter | 0.51 | 6.28 | < 0.001*** | 0.35 | 0.67 |
| Lactation Number | 0.25 | 6.42 | < 0.001*** | 0.17 | 0.32 |
| Year | 0.52 | 7.26 | < 0.001*** | 0.38 | 0.66 |
| Week Post Calving | 2.43 | 2.01 | 0.04* | 0.06 | 4.79 |
| Week of Gestation | -0.07 | -15.21 | < 0.001*** | -0.08 | -0.06 |
| Days in Milk | -0.34 | -1.96 | 0.05* | -0.68 | 0.00 |
| Net Energy (NE) | 0.03 | 58.97 | < 0.001*** | 0.03 | 0.03 |
| Weekly metabolic body weight (kg) | 0.02 | 19.12 | < 0.001*** | 0.01 | 0.02 |
| Average weekly weight change (kg) | 0.11 | 6.26 | < 0.001*** | 0.07 | 0.14 |

^1^Estimates represent regression coefficients (*β*) with associated z-values, *P*-values, and 95% confidence intervals.

^2^Significant covariates were marked * *P* < 0.05, ** *P* < 0.01, *** *P* < 0.001.

^3^Season effects are presented relative to Autumn (reference category).

Table S2: Summary of 190 SNP associated with Residual Feed Intake (RFI) using the Random Forest model^1^.

| SNP ID | rsID | Chromosome | Position (bp) | Associated Genes |
| --- | --- | --- | --- | --- |
| BTA-111131-no-rs | rs722025878 | 1 | 34584932 | No Genes Found |
| BovineHD0100009928 | rs383407972 | 1 | 34604373 | No Genes Found |
| ARS-BFGL-NGS-34687 | rs43671830 | 1 | 45900804 | IMPG2, SENP7, TRMT10C |
| BovineHD0100020551 | rs724102578 | 1 | 71658243 | DLG1, U6 |
| ARS-BFGL-NGS-78165 | rs721954380 | 1 | 72605572 | No Genes Found |
| ARS-BFGL-NGS-45476 | rs719439237 | 1 | 72640881 | No Genes Found |
| BTA-120764-no-rs | rs482521790 | 1 | 137284174 | ACP3, DNAJC13 |
| BovineHD0100040916 | rs451069627 | 1 | 142521116 | ABCG1, TFF2, TFF3 |
| ARS-BFGL-BAC-12960 | rs524967112 | 1 | 147812230 | No Genes Found |
| ARS-BFGL-NGS-35604 | rs518208565 | 1 | 150084135 | KCNJ6 |
| ARS-BFGL-NGS-16717 | rs454262895 | 2 | 970561 | CYFIP1, HERC2, NIPA1, NIPA2 |
| Hapmap40228-BTA-88147 | rs454077197 | 2 | 4720583 | AMMECR1L, POLR2D, SFT2D3, WDR33 |
| Hapmap51331-BTA-85692 | rs447911748 | 2 | 8272673 | No Genes Found |
| BTB-01435656 | rs519501612 | 2 | 8305740 | No Genes Found |
| ARS-BFGL-NGS-30075 | rs135526172 | 2 | 10560879 | FSIP2 |
| Hapmap39006-BTA-116188 | rs719635421 | 2 | 30481073 | GALNT3, TTC21B |
| BovineHD0200016373 | rs378850490 | 2 | 57728742 | No Genes Found |
| ARS-BFGL-NGS-10941 | rs452093519 | 3 | 16179998 | AQP10, ATP8B2, HAX1, IL6R, SHE, TDRD10, UBAP2L |
| ARS-BFGL-NGS-76744 | rs479439982 | 3 | 20652293 | BOLA1, H2AC18, H2AC20, H2BC21, MTMR11, OTUD7B, SF3B4, SV2A, VPS45 |
| ARS-BFGL-NGS-79371 | rs522945157 | 3 | 22550916 | FMO5, PRKAB2, U1 |
| ARS-BFGL-NGS-40236 | rs477174331 | 3 | 26496378 | CD2, IGSF3 |
| ARS-BFGL-NGS-36823 | rs481612548 | 3 | 26971936 | ATP1A1 |
| ARS-BFGL-NGS-109989 | rs210876813 | 3 | 53638272 | LRRC8B, LRRC8C |
| Hapmap43782-BTA-118016 | rs435160023 | 3 | 56602309 | LMO4 |
| BovineHD0300017852 | rs1115147822 | 3 | 59394905 | SSX2IP |
| BTB-01472286 | rs459189426 | 3 | 70399891 | TNNI3K |
| Hapmap48852-BTA-68859 | rs445570133 | 3 | 86467404 | FGGY |
| ARS-BFGL-NGS-116828 | rs466493450 | 3 | 93116911 | CPT2, CZIB, PODN, SCP2, SLC1A7 |
| INRA-431 | rs460739542 | 3 | 94250186 | BTF3L4, KTI12, RAB3B, TXNDC12, ZFYVE9 |
| ARS-BFGL-NGS-107522 | rs465051629 | 3 | 94558099 | NRDC, OSBPL9, bta-mir-761 |
| BTB-00147175 | rs462953852 | 3 | 96072837 | ELAVL4 |
| BTB-00147033 | rs721434867 | 3 | 96293488 | ELAVL4 |
| BTB-00146940 | rs445458564 | 3 | 96417242 | No Genes Found |
| BTB-01991408 | rs720139973 | 3 | 96545620 | No Genes Found |
| ARS-BFGL-NGS-90515 | rs434755059 | 3 | 96702795 | No Genes Found |
| ARS-BFGL-NGS-113457 | rs522923097 | 3 | 97975773 | SKINT1, SLC5A9, SPATA6 |
| ARS-BFGL-NGS-102345 | rs454396237 | 3 | 98085843 | SKINT1, SLC5A9 |
| ARS-BFGL-NGS-30298 | rs724067948 | 3 | 98123394 | SKINT1 |
| ARS-USMARC-Parent-DQ489377-rs29026932 | rs714166110 | 3 | 98188384 | SKINT1, TRABD2B, U6 |
| ARS-BFGL-NGS-110549 | rs110094849 | 3 | 98199726 | TRABD2B, U6 |
| ARS-BFGL-NGS-95604 | rs451114490 | 3 | 98284456 | TRABD2B, U6 |
| Hapmap47700-BTA-69019 | rs520980539 | 3 | 98370825 | TRABD2B |
| Hapmap50068-BTA-69023 | rs210641155 | 3 | 98450919 | TRABD2B |
| IDBv30300005627 | rs797458176 | 3 | 99939081 | LURAP1, P3R3URF, PIK3R3, POMGNT1, RAD54L, TSPAN1, U6 |
| ARS-BFGL-NGS-111078 | rs480306627 | 3 | 99941168 | LURAP1, P3R3URF, PIK3R3, POMGNT1, RAD54L, TSPAN1, U6 |
| BTB-00148969 | rs458869254 | 3 | 101854732 | DMAP1, ERI3, KLF17 |
| ARS-BFGL-NGS-117992 | rs452136635 | 3 | 102056606 | ARTN, ATP6V0B, B4GALT2, CCDC24, DPH2, IPO13, SLC6A9, ST3GAL3 |
| ARS-BFGL-NGS-35702 | rs526233028 | 4 | 5290218 | DDC, GRB10 |
| ARS-BFGL-NGS-23776 | rs452041955 | 4 | 5312990 | DDC, GRB10 |
| ARS-BFGL-NGS-5701 | rs520761527 | 4 | 5424181 | DDC, FIGNL1, GRB10, IKZF1 |
| BTB-00208066 | rs434309125 | 4 | 110740163 | CNTNAP2 |
| BTA-87036-no-rs | rs465055259 | 5 | 14741492 | LRRIQ1, TSPAN19, U6 |
| ARS-BFGL-NGS-114594 | rs721944421 | 5 | 22599252 | EEA1, PLEKHG7, U6 |
| Hapmap23146-BTA-123179 | rs211472081 | 5 | 22847985 | EEA1 |
| ARS-BFGL-NGS-113311 | - | 5 | 27633265 | KRT7, KRT80, KRT83, KRT86, KRT89 |
| IDBv20500000976 | rs5476263843 | 5 | 28615358 | BIN2, CSRNP2, DAZAP2, LETMD1, POU6F1, SMAGP, TFCP2 |
| ARS-BFGL-NGS-1251 | rs527218387 | 5 | 30639518 | DHH, LMBR1L, TUBA1A, TUBA1B, TUBA1C |
| BTB-00225243 | rs1116606869 | 5 | 36772262 | IRAK4, PUS7L |
| ARS-BFGL-NGS-101198 | rs208972594 | 5 | 77563893 | BICD1 |
| ARS-BFGL-NGS-820 | rs799630280 | 5 | 84058638 | No Genes Found |
| BovineHD0500023959 | rs439617687 | 5 | 84683137 | KRAS |
| Hapmap27529-BTC-050639 | rs465388283 | 6 | 39034201 | No Genes Found |
| BovineHD0600011456 | rs1115379768 | 6 | 42220411 | ADGRA3, GBA3 |
| Hapmap44513-BTA-107931 | rs462515228 | 6 | 43419974 | PPARGC1A |
| ARS-BFGL-NGS-24522 | rs461823475 | 6 | 87878364 | SNORD42 |
| BovineHD0700002394 | rs525573873 | 7 | 9219315 | OR7A79, OR7A83 |
| BovineHD0700003931 | rs208307970 | 7 | 14604352 | ANGPTL6, COL5A3, DNMT1, OLFM2, P2RY11, PPAN, RDH8, SHFL, SNORD105, SNORD105B |
| IDBv20700001547 | rs722548681 | 7 | 17360044 | OR2Z1 |
| BTA-110073-no-rs | rs378142165 | 7 | 27732801 | No Genes Found |
| BTB-00313229 | rs459967708 | 7 | 60088037 | HTR4 |
| Hapmap40463-BTA-79519 | rs715973262 | 7 | 63052639 | ATOX1, G3BP1, GLRA1 |
| ARS-BFGL-NGS-52705 | rs450815432 | 8 | 95468310 | FKTN, TAL2, TMEM38B, U6 |
| BTA-85169-no-rs | rs442185484 | 9 | 101286417 | MPC1, SFT2D1, U6 |
| ARS-BFGL-NGS-41356 | rs521263270 | 9 | 101966211 | CCR6, GPR31, TTLL2, U4, UNC93A |
| ARS-BFGL-NGS-5665 | rs723776491 | 10 | 43012833 | SOS2, VCPKMT, bta-mir-10171 |
| ARS-BFGL-NGS-109218 | rs524147803 | 10 | 45488421 | TRIP4, ZNF609 |
| ARS-BFGL-NGS-77250 | rs459864059 | 10 | 52814326 | CGNL1, MYZAP |
| BTB-01420453 | rs444229014 | 10 | 69287432 | OTX2 |
| Hapmap29285-BTA-162580 | rs386049531 | 10 | 69721690 | AP5M1, EXOC5, NAA30, SNORA72 |
| ARS-BFGL-NGS-118420 | rs133687261 | 11 | 6543992 | MAP4K4 |
| BTB-00455867 | rs722818831 | 11 | 8449441 | No Genes Found |
| ARS-BFGL-NGS-106748 | rs721224620 | 11 | 84974574 | U2 |
| ARS-BFGL-NGS-110235 | rs457904335 | 11 | 85367073 | No Genes Found |
| ARS-BFGL-NGS-113507 | rs462349605 | 11 | 98407974 | CDK9, CFAP157, CYCS, ENG, FPGS, PTRH1, SH2D3C, STXBP1, TOR2A, TTC16 |
| ARS-BFGL-NGS-112130 | rs519533811 | 11 | 102548124 | CFAP77, TTF1 |
| ARS-BFGL-NGS-25860 | rs799496048 | 12 | 18472407 | CYSLTR2 |
| ARS-BFGL-NGS-64184 | rs383790661 | 12 | 22372861 | No Genes Found |
| BTA-117645-no-rs | rs386072723 | 12 | 25216679 | CCNA1, SOHLH2, SPART |
| ARS-BFGL-BAC-15016 | rs469319576 | 12 | 29649126 | B3GLCT |
| ARS-BFGL-NGS-36563 | rs471705744 | 12 | 52003943 | ACOD1, KCTD12 |
| BTA-31832-no-rs | rs451244485 | 13 | 18950080 | PARD3 |
| Hapmap53719-rs29024306 | rs455774181 | 13 | 44493281 | No Genes Found |
| Hapmap41228-BTA-32897 | rs523543391 | 13 | 52531127 | EBF4, IDH3B, NOP56, SNORD110, SNORD56, SNORD57, SNORD86, TMC2 |
| ARS-BFGL-NGS-116740 | rs3423341312 | 13 | 65965727 | DNPH1, MTCL2, RBL1, SAMHD1, TLDC2 |
| ARS-BFGL-NGS-28898 | rs460829887 | 14 | 19576550 | MCM4, PRKDC, UBE2V2 |
| Hapmap47925-BTA-36060 | rs520446401 | 14 | 20273115 | SNTG1 |
| Hapmap23524-BTC-065402 | rs519367752 | 14 | 26874157 | CLVS1 |
| ARS-BFGL-NGS-52422 | rs438663325 | 14 | 39295276 | U6 |
| BTB-00569375 | rs521554427 | 14 | 49240465 | No Genes Found |
| BTB-00488528 | rs210078529 | 14 | 54586619 | KCNV1 |
| Hapmap60925-rs29018458 | rs800257552 | 14 | 55765964 | No Genes Found |
| BTA-98030-no-rs | rs465513404 | 14 | 56224870 | EIF3E |
| Hapmap25154-BTC-014295 | rs449134586 | 14 | 57541911 | No Genes Found |
| Hapmap50325-BTA-35116 | rs110940372 | 14 | 57728143 | ABRA |
| Hapmap47582-BTA-35120 | rs452170318 | 14 | 57785040 | ABRA, OXR1 |
| BTB-00752634 | rs720396166 | 14 | 59049305 | ZFPM2 |
| ARS-BFGL-BAC-26802 | rs209855676 | 14 | 59502755 | No Genes Found |
| ARS-BFGL-NGS-1405 | rs517773739 | 14 | 62508173 | NCALD |
| BTB-00574776 | rs447020177 | 14 | 63353353 | YWHAZ |
| ARS-BFGL-NGS-41976 | rs448700951 | 14 | 64968182 | VPS13B |
| Hapmap39171-BTA-35277 | rs483149016 | 14 | 66951519 | CPQ, TSPYL5, bta-mir-584-1 |
| UA-IFASA-5830 | rs135298101 | 14 | 67048933 | CPQ, bta-mir-584-1 |
| Hapmap39201-BTA-35312 | rs110562030 | 14 | 68304416 | No Genes Found |
| ARS-BFGL-NGS-3386 | rs379975924 | 14 | 68817604 | SNORA70 |
| ARS-BFGL-NGS-859 | rs714857302 | 14 | 73919098 | OSGIN2, RIPK2 |
| ARS-BFGL-NGS-112763 | rs463158958 | 14 | 75832105 | CNBD1, CNGB3 |
| UA-IFASA-7973 | rs135305594 | 14 | 76043148 | CNGB3, CPNE3 |
| BovineHD1400021668 | rs380668022 | 14 | 77280386 | CA13 |
| ARS-BFGL-NGS-114553 | rs385468033 | 15 | 8525251 | No Genes Found |
| BTB-00614032 | rs720587100 | 15 | 71013018 | No Genes Found |
| BovineHD1500023106 | rs460178467 | 15 | 79247462 | OR8J16, OR8J19, OR8J3F, OR8K1, OR8K3B, OR8K5B, OR8K61, OR8K64 |
| ARS-BFGL-NGS-69075 | rs526473164 | 15 | 80404264 | APLNR, LRRC55, TNKS1BP1 |
| ARS-BFGL-NGS-53886 | rs383946087 | 16 | 2005666 | ETNK2, GOLT1A, KISS1, PLEKHA6, REN, SOX13 |
| BTB-00640892 | rs519856135 | 16 | 45197527 | No Genes Found |
| ARS-BFGL-BAC-35338 | rs520122982 | 16 | 71773792 | LPGAT1, NEK2, SLC30A1 |
| ARS-BFGL-NGS-42222 | rs474771835 | 16 | 80127630 | PPP1R12B, SYT2 |
| Hapmap36186-SCAFFOLD206949_1268 | rs377957774 | 17 | 6556513 | PRSS48, SH3D19 |
| ARS-BFGL-NGS-30750 | rs446648080 | 18 | 8194300 | CMIP, bta-mir-2883 |
| ARS-BFGL-NGS-75672 | rs41885095 | 18 | 44392765 | No Genes Found |
| ARS-BFGL-NGS-54821 | rs518133581 | 18 | 47563494 | ZFP30, ZNF345, ZNF420, ZNF570 |
| ARS-BFGL-NGS-13393 | rs722290840 | 18 | 50616688 | B3GNT8, B9D2, BCKDHA, DMAC2, ERICH4, EXOSC5, TMEM91 |
| BovineHD1800014888 | - | 18 | 50629799 | B3GNT8, BCKDHA, DMAC2, ERICH4, EXOSC5 |
| Hapmap53843-rs29014653 | rs450296506 | 18 | 51939973 | CADM4, IRGC, KCNN4, LYPD5, PLAUR, SMG9, ZNF283 |
| BTA-44117-no-rs | rs449140388 | 18 | 64188147 | PEG3, bta-mir-2900 |
| BTB-00755453 | rs520823527 | 19 | 48007453 | CCDC47, DDX42, FTSJ3, LIMD2, MAP3K3, PSMC5, SMARCD2, STRADA, TACO1, TCAM1, bta-mir-10173 |
| ARS-BFGL-NGS-105192 | rs450552885 | 20 | 3456844 | No Genes Found |
| ARS-BFGL-NGS-17028 | rs444485716 | 20 | 7491951 | No Genes Found |
| Hapmap48634-BTA-118065 | rs1114342374 | 20 | 21107750 | No Genes Found |
| ARS-BFGL-NGS-29468 | rs463455275 | 20 | 37066726 | CPLANE1, NIPBL, NUP155, WDR70 |
| ARS-BFGL-NGS-111420 | rs109940209 | 20 | 37553988 | SLC1A3 |
| ARS-BFGL-NGS-114295 | rs437497121 | 20 | 41936114 | C20H5orf22, DROSHA, U2 |
| ARS-BFGL-NGS-7967 | rs380506790 | 20 | 58758614 | TRIO |
| ARS-BFGL-NGS-104203 | rs110434529 | 20 | 63185279 | No Genes Found |
| Hapmap60703-rs29010376 | rs378434094 | 21 | 42089249 | GPR33, NUBPL |
| Hapmap58214-rs29015775 | rs720708893 | 22 | 13203551 | EIF1B, ENTPD3, MYRIP, RPL14, ZNF619 |
| ARS-BFGL-NGS-15552 | rs443943114 | 22 | 15589177 | ABHD5, ANO10, SNORD36 |
| BTA-92840-no-rs | rs479685387 | 22 | 24219999 | CNTN4 |
| ARS-BFGL-NGS-20317 | rs722273832 | 22 | 29854903 | EIF4E3, GPR27, PROK2 |
| Hapmap47039-BTA-54432 | rs473254281 | 22 | 38877794 | CADPS, FEZF2 |
| ARS-BFGL-NGS-110889 | rs210123852 | 23 | 34339845 | U6 |
| BovineHD2300010235 | rs452563309 | 23 | 35054000 | PRP-VII, PRP3, PRP9, U6 |
| Hapmap42255-BTA-56826 | rs464126910 | 23 | 41313003 | DTNBP1 |
| ARS-BFGL-NGS-39445 | rs799772465 | 24 | 21071943 | C24H18orf21, ELP2, RPRD1A, SLC39A6 |
| ARS-BFGL-BAC-43578 | rs378365360 | 25 | 1864761 | ABCA3, CCNF, DNASE1L2, ECI1, RNPS1, bta-mir-940 |
| UA-IFASA-7169 | rs110247513 | 25 | 16647252 | CLEC19A, SYT17 |
| ARS-BFGL-NGS-3834 | rs432377741 | 25 | 18206998 | ACSM1, ACSM2B, ACSM5 |
| Hapmap47132-BTA-91548 | rs442700710 | 25 | 40730047 | AMZ1, BRAT1, CHST12, GRIFIN, IQCE, LFNG, TTYH3 |
| ARS-BFGL-NGS-43507 | rs474143957 | 26 | 7761759 | PRKG1 |
| Hapmap59335-rs29016866 | rs42089620 | 26 | 10689379 | ACTA2, FAS, STAMBPL1 |
| Hapmap41595-BTA-60800 | rs721467497 | 26 | 16791783 | CYP2C282, PDLIM1, SORBS1 |
| Hapmap58930-rs29010490 | rs466445109 | 26 | 16822073 | PDLIM1, SORBS1 |
| BTB-00930925 | rs715375637 | 26 | 20474308 | CNNM1, GOT1, NKX2-3, U6 |
| Hapmap35702-SCAFFOLD240765_1255 | rs379576777 | 26 | 20668949 | ABCC2, COX15, CUTC, ENTPD7, SLC25A28 |
| Hapmap46411-BTA-15820 | rs438787708 | 26 | 20984335 | CPN1, CYP2C23, DNMBP, ERLIN1, U6 |
| BTB-00932332 | rs524961352 | 26 | 22118554 | BTRC, LBX1 |
| ARS-BFGL-NGS-111090 | rs451639800 | 26 | 23920913 | ATP5MK, INA, PCGF6, PDCD11, TAF5, bta-mir-1307 |
| Hapmap42608-BTA-62411 | rs5408007005 | 27 | 19301092 | ASAH1, FGL1, PCM1 |
| BovineHD2700006562 | rs382129834 | 27 | 23350684 | C27H8orf48 |
| Hapmap44432-BTA-97711 | rs799429064 | 27 | 28298862 | No Genes Found |
| IDBv32700009883 | rs483073038 | 27 | 28331626 | NRG1 |
| IDBv32700009890 | rs481013208 | 27 | 28332168 | NRG1 |
| BTB-01796032 | rs715321181 | 27 | 31337533 | UNC5D |
| ARS-BFGL-NGS-115354 | rs469226642 | 27 | 31426001 | UNC5D |
| ARS-BFGL-NGS-75049 | rs385015104 | 28 | 3504349 | C28H1orf198 |
| BovineHD2800001779 | rs715986276 | 28 | 5996630 | PCNX2 |
| ARS-BFGL-NGS-10882 | rs522366551 | 28 | 7375756 | IRF2BP2 |
| ARS-BFGL-NGS-3616 | rs479796048 | 28 | 10511817 | RYR2 |
| BTA-104046-no-rs | rs523826733 | 28 | 23385803 | CTNNA3, LRRTM3 |
| BTB-00982413 | rs717465595 | 28 | 23829443 | CTNNA3 |
| BTB-01381791 | rs476030582 | 29 | 20199725 | LUZP2 |
| UA-IFASA-9601 | rs135683476 | 29 | 22660543 | ANO5 |
| ARS-BFGL-NGS-118751 | rs379401226 | 29 | 45974645 | LRP5, PPP6R3 |
| Hapmap41472-BTA-19981 | - | 30 | 24286879 | No Genes Found |
| ARS-BFGL-NGS-114657 | rs455983007 | 30 | 58788015 | AMMECR1, RTL9 |
| BovineHD3000025418 | rs461109361 | 30 | 92359410 | MAGED2, PFKFB1 |
| ARS-BFGL-NGS-36290 | rs452791158 | 30 | 108208784 | No Genes Found |
| BTA-95868-no-rs | rs477357085 | 30 | 108243497 | No Genes Found |
| ARS-BFGL-NGS-6742 | rs5477554796 | 30 | 121434178 | MBTPS2, SMPX, SMS |
| BovineHD3000041083 | - | 30 | 141891226 | No Genes Found |
| ARS-BFGL-NGS-113130 | - | 30 | 145726589 | No Genes Found |

^1^The table lists significant Single Nucleotide Polymorphisms (SNP) identified in the study. For each SNP, the table provides the unique identifier (SNP ID and rsID), chromosomal location (Chromosome and Position), and associated genes (Total: 373 unique genes).

Table S3: Summary of 473 SNP associated with Residual Feed Intake (RFI) using the Multi-Layer Perceptron model^1^.

| SNP ID | rsID | Chromosome | Position (bp) | Associated Genes |
| --- | --- | --- | --- | --- |
| ARS-BFGL-NGS-29653 | rs384558022 | 1 | 2049400 | DNAJC28, DONSON, GART, IFNGR2, SON, TMEM50B |
| BovineHD0100001969 | rs517320542 | 1 | 6025840 | GRIK1 |
| ARS-BFGL-NGS-32422 | rs380649324 | 1 | 15937076 | NCAM2 |
| BTA-118719-no-rs | rs384959446 | 1 | 16673009 | No Genes Found |
| BTB-01511592 | rs210214544 | 1 | 18028700 | No Genes Found |
| BTA-120720-no-rs | rs42277071 | 1 | 22407486 | No Genes Found |
| BTB-00010474 | rs524606807 | 1 | 24757983 | ROBO2 |
| ARS-BFGL-NGS-11151 | rs444024538 | 1 | 26425477 | ROBO1 |
| BTB-01303185 | rs210359712 | 1 | 51563701 | No Genes Found |
| ARS-BFGL-NGS-89588 | rs526986207 | 1 | 52834285 | CD47, IFT57 |
| BovineHD0100015080 | rs463564445 | 1 | 53595299 | TRAT1 |
| ARS-BFGL-NGS-43783 | rs461187129 | 1 | 54774488 | No Genes Found |
| ARS-BFGL-NGS-114627 | rs1116584525 | 1 | 74355653 | No Genes Found |
| ARS-BFGL-NGS-34525 | rs478150750 | 1 | 75588102 | No Genes Found |
| ARS-BFGL-NGS-112470 | rs5361660125 | 1 | 88489434 | No Genes Found |
| BTB-00006833 | rs443845423 | 1 | 103623793 | No Genes Found |
| BTB-00049653 | rs472933896 | 1 | 107910690 | IQCJ |
| BTB-00738246 | rs516939158 | 1 | 112769407 | No Genes Found |
| ARS-BFGL-BAC-7227 | rs379636519 | 1 | 126931293 | TFDP2 |
| BTB-00056233 | rs433607348 | 1 | 127615037 | PXYLP1, ZBTB38 |
| BovineHD0100037755 | rs5363663001 | 1 | 132566579 | STAG1 |
| BovineHD0100039570 | rs210756446 | 1 | 138601259 | NEK11, NUDT16, U6 |
| ARS-BFGL-NGS-103594 | rs441668211 | 1 | 149043397 | HLCS, SIM2 |
| BTA-116423-no-rs | rs723379413 | 1 | 151736540 | COL6A5, COL6A6 |
| ARS-BFGL-NGS-11586 | rs455006880 | 1 | 153045653 | bta-mir-2285ao-2 |
| ARS-BFGL-NGS-40247 | rs453278671 | 1 | 154133697 | PLCL2, TBC1D5 |
| BTB-01739851 | rs516569708 | 2 | 4650209 | AMMECR1L, POLR2D, WDR33 |
| ARS-BFGL-NGS-24135 | rs461920625 | 2 | 5193223 | ERCC3, MAP3K2, PROC, bta-mir-2285aa |
| ARS-BFGL-NGS-4516 | rs522129324 | 2 | 6151365 | C2H2orf88, HIBCH |
| BTA-27415-no-rs | rs716771356 | 2 | 7751590 | GULP1, U6 |
| BovineHD0200006924 | rs435278290 | 2 | 24211998 | ITGA6, bta-mir-2352 |
| BTB-00091700 | rs478965842 | 2 | 26308838 | MYO3B, U6, UBR3, bta-mir-12020 |
| ARS-BFGL-BAC-33842 | rs438144890 | 2 | 31050929 | SCN2A, SCN3A, U6 |
| BTB-01944931 | rs458299999 | 2 | 31500118 | COBLL1, U6, bta-mir-11978 |
| BovineHD0200009397 | rs451705910 | 2 | 31700760 | GRB14 |
| ARS-BFGL-NGS-34549 | rs134980268 | 2 | 38340255 | U6, UPP2 |
| BTA-53248-no-rs | rs466742182 | 2 | 64549576 | NCKAP5 |
| ARS-BFGL-NGS-80573 | rs447537133 | 2 | 68089041 | No Genes Found |
| BTA-101689-no-rs | rs720071559 | 2 | 68508316 | No Genes Found |
| BTB-01831918 | rs385269011 | 2 | 68849454 | No Genes Found |
| BTB-00121016 | rs478255879 | 2 | 69359925 | No Genes Found |
| ARS-BFGL-NGS-86652 | rs210713282 | 2 | 72790595 | GLI2 |
| BTB-00110220 | rs110082963 | 2 | 110332434 | PAX3, bta-mir-2284y-5 |
| BTB-00110046 | rs477702983 | 2 | 110477897 | PAX3, RNF228, bta-mir-2284y-5 |
| ARS-BFGL-NGS-22422 | rs523557501 | 2 | 123421577 | No Genes Found |
| ARS-BFGL-NGS-102755 | rs42490559 | 2 | 135347580 | PADI1, PADI3, PADI4, PADI6 |
| BovineHD0300000312 | rs441494854 | 3 | 1213032 | CD247, CREG1, POU2F1 |
| BovineHD0300002659 | rs526950723 | 3 | 8150471 | CFAP126, MPZ, PCP4L1, SDHC |
| BovineHD0300002694 | rs448721241 | 3 | 8234591 | ADAMTS4, APOA2, B4GALT3, CFAP126, FCER1G, MPZ, NDUFS2, NR1I3, PCP4L1, PPOX, SDHC, TOMM40L, USP21 |
| INRA-304 | rs437616591 | 3 | 9579325 | ATP1A2, ATP1A4, CASQ1, IGSF8, KCNJ10, KCNJ9, PIGM |
| BTA-88149-no-rs | rs482622087 | 3 | 34421834 | CLCC1, GPSM2, RNaseP_nuc, SNORA70, WDR47 |
| BTB-01580562 | rs379882219 | 3 | 65684294 | ADGRL4 |
| BTB-01749645 | rs471945155 | 3 | 68822799 | ST6GALNAC3, U6 |
| BTB-01392993 | rs446958575 | 3 | 71596348 | No Genes Found |
| INRA-321 | rs43346004 | 3 | 81120497 | CACHD1 |
| BTB-01240613 | rs467905241 | 3 | 85984517 | CYP2J30 |
| ARS-BFGL-NGS-57301 | rs449409455 | 3 | 116378417 | No Genes Found |
| ARS-BFGL-NGS-3169 | rs110555808 | 3 | 116694369 | COL6A3 |
| BovineHD0400003059 | rs383680763 | 4 | 9985874 | CDK6, FAM133B |
| ARS-BFGL-NGS-117720 | rs1117077083 | 4 | 14349978 | DLX5, DLX6 |
| IDBv30400005683 | rs133754838 | 4 | 15052550 | ASNS, TAC1 |
| BovineHD0400004572 | rs447254234 | 4 | 15366313 | ASNS, C1GALT1, COL28A1 |
| ARS-BFGL-NGS-35546 | rs109697274 | 4 | 15468000 | COL28A1 |
| BTA-72744-no-rs | rs109329471 | 4 | 27203687 | HDAC9 |
| ARS-BFGL-NGS-72533 | rs135318998 | 4 | 27414604 | HDAC9 |
| ARS-BFGL-NGS-48336 | rs1117104917 | 4 | 30308043 | DNAH11, SP4 |
| BTB-01934112 | rs136845951 | 4 | 34059453 | No Genes Found |
| BTB-01771861 | rs110525265 | 4 | 42257334 | MAGI2 |
| BTA-70725-no-rs | rs461703241 | 4 | 57809364 | IMMP2L |
| ARS-BFGL-NGS-100947 | rs444069050 | 4 | 61444963 | HERPUD2 |
| ARS-BFGL-NGS-6754 | rs451851418 | 4 | 71089406 | GSDME, OSBPL3, PALS2 |
| ARS-BFGL-NGS-21842 | rs380004547 | 4 | 73531264 | 5S_rRNA |
| BTB-01328019 | rs715789928 | 4 | 74234970 | No Genes Found |
| BovineHD0400020977 | rs207862256 | 4 | 75810034 | No Genes Found |
| BTB-00195629 | rs438664540 | 4 | 77954275 | HECW1 |
| BTB-01162427 | rs798329361 | 4 | 81021023 | CDK13, MPLKIP, SUGCT |
| BovineHD0400026170 | rs5476237023 | 4 | 93672302 | NRF1, UBE2H |
| BTA-71670-no-rs | rs800504774 | 4 | 95465677 | No Genes Found |
| BovineHD0400031386 | rs133396331 | 4 | 109675171 | CNTNAP2 |
| ARS-BFGL-NGS-33589 | rs446543999 | 4 | 112815065 | ATP6V0E2, GIMAP7, GIMAP8, LRRC61, RARRES2, REPIN1, ZNF775 |
| ARS-BFGL-NGS-17799 | rs136160221 | 4 | 118873541 | PTPRN2 |
| BTB-00216290 | rs110316882 | 5 | 5240327 | No Genes Found |
| ARS-BFGL-NGS-68464 | rs480115567 | 5 | 16163636 | No Genes Found |
| BovineHD0500006566 | rs438543278 | 5 | 22676543 | EEA1, PLEKHG7, U6 |
| ARS-BFGL-NGS-50553 | rs526255110 | 5 | 26181410 | HOXC13, U6 |
| ARS-BFGL-NGS-15960 | rs208470450 | 5 | 28024945 | ACVRL1, ANKRD33, FIGNL2, SCN8A, TMDD1 |
| ARS-BFGL-NGS-25578 | rs464619144 | 5 | 28120980 | FIGNL2, SCN8A, TMDD1 |
| ARS-USMARC-635 | rs451457425 | 5 | 30353509 | KCNH3, MCRS1, SPATS2 |
| BovineHD0500008963 | rs434526592 | 5 | 30635318 | DHH, LMBR1L, TUBA1A, TUBA1B, TUBA1C |
| ARS-BFGL-NGS-53461 | rs437367941 | 5 | 30659497 | DHH, LMBR1L, RHEBL1, TUBA1A, TUBA1B, TUBA1C |
| ARS-BFGL-NGS-43909 | rs464954745 | 5 | 30879776 | ARF3, CACNB3, CCDC65, DDN, DDX23, FKBP11, KMT2D, PRKAG1, RND1, WNT1, WNT10B, bta-mir-2426 |
| ARS-BFGL-NGS-14419 | rs464047503 | 5 | 32502917 | ENDOU, HDAC7, RAPGEF3, RPAP3, SLC48A1, VDR |
| ARS-BFGL-NGS-40375 | rs800227365 | 5 | 36041533 | TMEM117 |
| ARS-BFGL-NGS-66566 | rs472366336 | 5 | 36122701 | TMEM117 |
| BTB-01832706 | rs440051679 | 5 | 37331234 | No Genes Found |
| BTB-01812364 | rs460501607 | 5 | 41666974 | ABCD2, KIF21A, bta-mir-2428 |
| BTB-01255969 | rs523269796 | 5 | 42743482 | 5S_rRNA, PTPRR |
| ARS-BFGL-NGS-38733 | rs1114529231 | 5 | 43940765 | CCT2, FRS2, LRRC10, bta-mir-2427 |
| BTB-00226565 | rs381618062 | 5 | 44009723 | FRS2, YEATS4 |
| ARS-BFGL-NGS-7433 | rs469487178 | 5 | 44885503 | CPM, MDM2 |
| ARS-BFGL-NGS-104108 | rs433417772 | 5 | 66480185 | U6 |
| IDBv20500001024 | rs137060805 | 5 | 66530231 | PAH, U6 |
| IDBv30500005979 | rs5476305772 | 5 | 75266801 | CSF2RB, NCF4, PVALB |
| BTB-00232546 | rs434387809 | 5 | 77311671 | FGD4, U6 |
| ARS-BFGL-NGS-106744 | rs438464078 | 5 | 77780682 | BICD1 |
| BovineHD0500022233 | rs208532163 | 5 | 78340433 | AMN1, DENND5B, ETFBKMT, U6 |
| ARS-BFGL-NGS-92011 | rs477810544 | 5 | 80194437 | ERGIC2, FAR2, OVCH1, TMTC1 |
| ARS-BFGL-NGS-88309 | rs801239169 | 5 | 98618390 | BOTA-T2R10B, SMIM10L1, T2R12, T2R65A, TAS2R42, TAS2R46 |
| ARS-BFGL-NGS-114092 | rs377971450 | 5 | 100493514 | KLRB1, U6 |
| ARS-BFGL-NGS-25950 | rs801395062 | 5 | 102050795 | WC-7 |
| ARS-BFGL-NGS-40068 | rs1116677877 | 5 | 110043735 | CSNK1E, KCNJ4, MAFF, PLA2G6, TMEM184B |
| ARS-BFGL-NGS-32908 | rs5476331886 | 5 | 110320607 | CBY1, DMC1, DNAL4, FAM227A, GTPBP1, JOSD1, SUN2, TOMM22, U6 |
| BovineHD0500032070 | rs210396694 | 5 | 111024410 | ATF4, CACNA1I, MIEF1, RPS19BP1 |
| ARS-BFGL-NGS-86565 | rs384728562 | 5 | 111420622 | FAM83F, GRAP2, TNRC6B |
| ARS-BFGL-NGS-10616 | rs454723786 | 5 | 112494968 | ACO2, PHF5A, RANGAP1, TEF, TOB2, U6, ZC3H7B |
| IDBv30500006065 | rs5476334310 | 5 | 112636761 | ACO2, CSDC2, DESI1, PHF5A, PMM1, POLR3H, SNU13, TOB2, XRCC6 |
| ARS-BFGL-NGS-3932 | - | 5 | 121028451 | No Genes Found |
| ARS-BFGL-NGS-38440 | - | 5 | 121062420 | No Genes Found |
| ARS-BFGL-NGS-107847 | rs459869785 | 6 | 7614521 | No Genes Found |
| BovineHD0600004364 | rs477371213 | 6 | 16145912 | COL25A1 |
| BTB-01038669 | rs522518259 | 6 | 19035074 | TBCK |
| ARS-BFGL-NGS-22019 | rs797993687 | 6 | 26559101 | STPG2 |
| ARS-BFGL-NGS-7249 | rs469875488 | 6 | 30050205 | PDLIM5, Y_RNA |
| ARS-BFGL-NGS-75543 | rs801084179 | 6 | 58638088 | KLB, LIAS, RFC1, RPL9, SMIM14, UGDH |
| BTB-01601425 | rs470190051 | 6 | 66235480 | ATP10D, CORIN |
| IDBv30600006207 | rs461360061 | 6 | 66316229 | ATP10D, CORIN |
| BovineHD0600030162 | rs717033902 | 6 | 107202541 | No Genes Found |
| ARS-BFGL-NGS-32423 | rs722618187 | 7 | 5955666 | HAUS8, MYO9B, NR2F6, OCEL1, U6, USE1, USHBP1 |
| IDBv20700001518 | rs5391823032 | 7 | 8547758 | OR7A107, OR7A117, OR7A93, OR7C71 |
| BovineHD0700002649 | rs1116486346 | 7 | 10099512 | OR7A80, OR7A82, OR7A88 |
| BovineHD0700003100 | rs462715593 | 7 | 11869116 | BRME1, CC2D1A, MRI1, NANOS3, YJU2B, ZSWIM4, bta-mir-23a, bta-mir-24-2, bta-mir-27a |
| ARS-BFGL-NGS-111041 | rs380661956 | 7 | 40701860 | NLRP3, OR2B11, OR2H10, OR2W53, U6, ZNF496 |
| ARS-BFGL-NGS-18096 | rs480251694 | 7 | 55453345 | KCTD16, U6 |
| ARS-BFGL-NGS-107103 | rs716079631 | 7 | 57554684 | POU4F3, RBM27, TCERG1, U6 |
| BTA-79386-no-rs | rs800804867 | 7 | 63519073 | No Genes Found |
| ARS-BFGL-NGS-109962 | rs479651550 | 7 | 64022666 | No Genes Found |
| BTB-00316650 | rs452563135 | 7 | 65098028 | GRIA1 |
| BTB-01121435 | rs433254489 | 7 | 68666448 | No Genes Found |
| BTB-00319733 | rs454145040 | 7 | 75630151 | No Genes Found |
| ARS-BFGL-NGS-35829 | rs461989000 | 7 | 76199889 | No Genes Found |
| BTA-113319-no-rs | rs211160700 | 7 | 76828674 | No Genes Found |
| ARS-BFGL-NGS-31863 | rs450834638 | 7 | 79624430 | TENM2 |
| ARS-BFGL-NGS-12785 | rs136108620 | 7 | 82241276 | ATG10, ATP6AP1L, RPS23 |
| ARS-BFGL-NGS-36740 | rs469835647 | 7 | 82784340 | U6 |
| BTB-01503564 | rs211187195 | 7 | 85387851 | No Genes Found |
| ARS-BFGL-NGS-92532 | - | 7 | 86485159 | No Genes Found |
| BTA-88722-no-rs | rs466365292 | 7 | 98222585 | CHD1 |
| ARS-BFGL-NGS-100601 | rs465752937 | 7 | 99130598 | No Genes Found |
| ARS-BFGL-NGS-7009 | rs468183197 | 8 | 1406173 | NEK1, SH3RF1, U6 |
| BTA-81513-no-rs | rs383708719 | 8 | 5526186 | GALNT7, GALNTL6 |
| ARS-BFGL-NGS-118457 | rs442896464 | 8 | 6687056 | CEP44, FBXO8, U1 |
| BTA-81086-no-rs | rs518515543 | 8 | 7199643 | ADAM29, GLRA3, U2 |
| ARS-BFGL-NGS-93716 | rs460647629 | 8 | 15395242 | No Genes Found |
| BTB-00095699 | rs798806673 | 8 | 15534805 | LINGO2 |
| BTB-01925657 | rs526937154 | 8 | 15629883 | LINGO2 |
| BovineHD0800006644 | rs722174541 | 8 | 21860463 | No Genes Found |
| ARS-BFGL-NGS-108956 | rs469474271 | 8 | 33216307 | U1 |
| BTB-01184997 | rs431944860 | 8 | 34367019 | No Genes Found |
| BTB-02056135 | rs384250059 | 8 | 35398490 | No Genes Found |
| ARS-BFGL-NGS-68744 | rs799139304 | 8 | 37985778 | KDM4C |
| BTB-01838786 | rs478221737 | 8 | 43163468 | No Genes Found |
| ARS-BFGL-NGS-87235 | rs718060719 | 8 | 49037615 | ALDH1A1, TMC1 |
| ARS-BFGL-NGS-17993 | rs110397899 | 8 | 53216502 | VPS13A |
| BTB-00631715 | rs110448831 | 8 | 53743552 | GNA14, GNAQ |
| BovineHD0800022409 | rs447770837 | 8 | 74884485 | B4GALT1, DNAJA1, SMU1, bta-mir-12061 |
| ARS-BFGL-NGS-58677 | rs1114513695 | 8 | 85329708 | BARX1, PTPDC1 |
| ARS-BFGL-NGS-11414 | rs523264246 | 8 | 86690521 | AUH |
| ARS-BFGL-NGS-61328 | rs5520710416 | 8 | 86810388 | No Genes Found |
| ARS-BFGL-NGS-103332 | rs445439727 | 8 | 104340545 | TNC |
| ARS-BFGL-NGS-92824 | rs5376287288 | 8 | 111713971 | No Genes Found |
| ARS-BFGL-NGS-102553 | rs5377114431 | 8 | 112966306 | FAM110C |
| BovineHD0900002534 | rs136613312 | 9 | 10370048 | OGFRL1 |
| BovineHD0900002637 | rs717784622 | 9 | 10608795 | bta-mir-30a |
| BovineHD0900002643 | rs379304110 | 9 | 10628675 | bta-mir-30a |
| ARS-BFGL-NGS-110691 | rs1116872204 | 9 | 10726411 | No Genes Found |
| BovineHD0900002695 | rs110486913 | 9 | 10839055 | No Genes Found |
| BovineHD0900002717 | rs447749880 | 9 | 10949242 | No Genes Found |
| BovineHD0900002730 | rs714437802 | 9 | 10970517 | No Genes Found |
| ARS-BFGL-NGS-59162 | rs522906219 | 9 | 12707144 | DDX43, KCNQ5, KHDC3L, OOEP |
| ARS-BFGL-NGS-31200 | rs1116891916 | 9 | 13835430 | No Genes Found |
| BTB-01233147 | rs714897320 | 9 | 14194506 | No Genes Found |
| ARS-BFGL-NGS-14740 | rs722703814 | 9 | 14436985 | No Genes Found |
| BTB-01756385 | rs209336145 | 9 | 14642429 | COL12A1 |
| ARS-BFGL-NGS-14739 | rs516220986 | 9 | 15417248 | MYO6, SENP6 |
| ARS-BFGL-NGS-115705 | rs525887287 | 9 | 19040720 | LCA5 |
| BTB-01362120 | rs1114448176 | 9 | 24079580 | ECHDC1, RNF146, RSPO3 |
| ARS-BFGL-NGS-4479 | rs42233171 | 9 | 24272399 | RSPO3 |
| BTA-95806-no-rs | rs464511409 | 9 | 27272926 | NKAIN2 |
| BTB-01534273 | rs519007216 | 9 | 27741354 | TRDN |
| BTB-01887905 | rs464293961 | 9 | 30006014 | TBC1D32 |
| BTB-02047616 | rs482852594 | 9 | 31394607 | No Genes Found |
| ARS-BFGL-NGS-61170 | rs382006627 | 9 | 45121053 | HACE1, LIN28B |
| BTA-83597-no-rs | rs436518668 | 9 | 46632366 | No Genes Found |
| BTB-01215359 | rs433245287 | 9 | 53355112 | UFL1 |
| BTB-00393938 | rs381070225 | 9 | 54690915 | MANEA |
| ARS-BFGL-NGS-38956 | rs719364722 | 9 | 60033566 | BACH2 |
| BTB-00396392 | rs433813821 | 9 | 60837285 | ANKRD6, GABRR1, RRAGD, U6, UBE2J1, bta-mir-2903 |
| BTB-00396747 | rs481074239 | 9 | 61292190 | RNGTT |
| ARS-BFGL-NGS-34134 | rs136666859 | 9 | 66124313 | PTPRK |
| IDBv30900006778 | rs716541579 | 9 | 66129136 | PTPRK |
| BovineHD0900018328 | rs462744462 | 9 | 66428571 | PTPRK, U6 |
| BTA-97754-no-rs | rs480867878 | 9 | 71021904 | RPS12, SLC18B1 |
| BTA-24107-no-rs | rs446772956 | 9 | 95017314 | DYNLT1, SYTL3, TMEM181, TULP4 |
| ARS-BFGL-NGS-38971 | rs719213912 | 9 | 98306160 | PACRG |
| ARS-BFGL-NGS-109783 | rs135838657 | 9 | 100138190 | No Genes Found |
| ARS-BFGL-NGS-72792 | rs1116182726 | 9 | 103411929 | C9H6orf120, PHF10, WDR27 |
| BovineHD1000001480 | rs800995342 | 10 | 4854061 | AP3S1, ARL14EPL, LVRN |
| ARS-BFGL-NGS-53960 | rs110413909 | 10 | 6669494 | ANKRD31, HMGCR |
| ARS-BFGL-NGS-114232 | rs461283891 | 10 | 16548361 | No Genes Found |
| ARS-BFGL-NGS-99693 | rs472143184 | 10 | 17867003 | LRRC49, THSD4 |
| ARS-BFGL-NGS-28483 | rs473856809 | 10 | 21571180 | BCL2L2, CMTM5, EFS, HOMEZ, IL25, MYH6, MYH7, PABPN1, PPP1R3E, RNF212B, SLC22A17 |
| BTA-61235-no-rs | rs722202171 | 10 | 21698931 | ACIN1, BCL2L2, CEBPE, HOMEZ, PPP1R3E, RNF212B, SLC7A8, U3, U6 |
| ARS-BFGL-NGS-42815 | rs385869897 | 10 | 26054054 | ARHGEF40, METTL17, NDRG2, RNASE13, SLC39A2, TMEM253, TPPP2, ZNF219 |
| ARS-BFGL-NGS-111228 | rs209889779 | 10 | 26325867 | ANG, ANG2, BRB, RAM2, RNASE1, RNASE4, RNASE6 |
| ARS-BFGL-NGS-21988 | rs5389970402 | 10 | 47842705 | TLN2 |
| BovineHD1000021409 | rs433593264 | 10 | 75312006 | KCNH5 |
| ARS-BFGL-NGS-3286 | rs110981680 | 10 | 75448590 | RHOJ |
| ARS-BFGL-NGS-110760 | rs476634654 | 10 | 90172460 | NRXN3 |
| ARS-BFGL-NGS-33429 | rs715446894 | 10 | 93923726 | No Genes Found |
| BovineHD1000027425 | rs720658355 | 10 | 94963453 | No Genes Found |
| BTB-01311849 | rs519244360 | 10 | 95608495 | No Genes Found |
| BovineHD1100000217 | rs132857783 | 11 | 680024 | ANAPC1 |
| BovineHD1100000516 | rs516191183 | 11 | 1500078 | ACOXL, BUB1 |
| UA-IFASA-2313 | rs433036764 | 11 | 10604234 | BOLA3, DGUOK, TET3 |
| BTB-00461599 | rs477318073 | 11 | 13681749 | ADD2, CLEC4F, FIGLA |
| ARS-BFGL-NGS-40996 | rs730568687 | 11 | 19861225 | QPCT, U6 |
| ARS-BFGL-NGS-43804 | rs451020977 | 11 | 22588525 | SLC8A1 |
| BovineHD1100007890 | - | 11 | 26450110 | U6 |
| ARS-BFGL-NGS-15269 | rs523312154 | 11 | 29578421 | CALM2, STPG4, TTC7A |
| BovineHD1100011738 | rs1115626532 | 11 | 39760460 | No Genes Found |
| ARS-BFGL-NGS-112026 | rs1115606502 | 11 | 39936968 | No Genes Found |
| ARS-BFGL-NGS-91269 | rs800304856 | 11 | 45470166 | ST6GAL2 |
| ARS-BFGL-NGS-90404 | rs5378396481 | 11 | 49242330 | GNLY, NK1, NK2B, SFTPB |
| BTA-104617-no-rs | rs525364627 | 11 | 49824565 | TCF7L1 |
| ARS-BFGL-NGS-11740 | rs5378567105 | 11 | 54193489 | No Genes Found |
| BTB-01696272 | rs525876124 | 11 | 57184891 | No Genes Found |
| ARS-BFGL-NGS-17731 | rs1115266650 | 11 | 86926025 | ATP6V1C2, NOL10, PDIA6 |
| ARS-BFGL-NGS-28030 | rs720926082 | 11 | 89338824 | bta-mir-2284aa-1 |
| ARS-BFGL-NGS-44432 | rs132726490 | 11 | 104117370 | ABO, AGPAT2, DIPK1B, EGFL7, U6, bta-mir-126 |
| ARS-BFGL-NGS-100020 | rs517498813 | 11 | 104790820 | BRD3, BRD3OS, VAV2, WDR5 |
| ARS-BFGL-BAC-7349 | rs1117308365 | 12 | 8839027 | No Genes Found |
| BovineHD1200003278 | rs379016290 | 12 | 11419755 | KBTBD6, KBTBD7, MTRF1, NAA16 |
| ARS-BFGL-BAC-12468 | rs453040825 | 12 | 27577957 | STARD13 |
| ARS-BFGL-NGS-45371 | rs1116856915 | 12 | 27807391 | STARD13 |
| ARS-BFGL-NGS-32430 | rs5342370856 | 12 | 28487284 | N4BP2L2, PDS5B |
| ARS-BFGL-NGS-76809 | rs465304345 | 12 | 29238096 | RXFP2 |
| ARS-BFGL-NGS-94762 | rs443503605 | 12 | 29324327 | RXFP2 |
| BTA-83050-no-rs | rs109704398 | 12 | 29562851 | B3GLCT |
| ARS-BFGL-NGS-57685 | rs5342269500 | 12 | 29831757 | B3GLCT, HSPH1 |
| ARS-BFGL-NGS-34779 | rs516959300 | 12 | 30146703 | ALOX5AP, USPL1 |
| ARS-BFGL-NGS-57156 | rs5476756449 | 12 | 30992759 | MTUS2, SLC7A1 |
| ARS-BFGL-NGS-107176 | rs476361292 | 12 | 33779590 | ATP8A2, U6 |
| ARS-BFGL-NGS-59161 | rs519119779 | 12 | 37322461 | No Genes Found |
| ARS-BFGL-NGS-110832 | rs210534324 | 12 | 40009119 | PCDH9 |
| ARS-BFGL-NGS-115420 | rs110992419 | 12 | 50155758 | No Genes Found |
| BTB-00495763 | rs526722815 | 12 | 51864752 | No Genes Found |
| BTB-00495415 | rs516273825 | 12 | 52098655 | ACOD1, CLN5, FBXL3, KCTD12, MYCBP2 |
| BTB-00493134 | rs5343116352 | 12 | 52492536 | MYCBP2 |
| ARS-BFGL-NGS-34948 | rs208624050 | 12 | 53080458 | EDNRB |
| BTB-00496806 | rs476528072 | 12 | 53960037 | No Genes Found |
| BTB-00498136 | rs483246645 | 12 | 53986983 | No Genes Found |
| ARS-BFGL-NGS-103418 | rs798508989 | 12 | 59921146 | No Genes Found |
| BTA-65033-no-rs | rs721519633 | 12 | 61073334 | No Genes Found |
| BTB-00500246 | rs381444398 | 12 | 61153197 | HTATSF1 |
| ARS-BFGL-NGS-57684 | rs5343546189 | 12 | 62266267 | SLITRK5 |
| BTA-85739-no-rs | rs523845273 | 12 | 62307557 | SLITRK5 |
| BovineHD1200026120 | - | 12 | 89220228 | No Genes Found |
| ARS-BFGL-NGS-116758 | rs801003005 | 13 | 5827434 | No Genes Found |
| ARS-BFGL-NGS-13253 | rs521650659 | 13 | 10194150 | KIF16B |
| BTA-102427-no-rs | rs460080020 | 13 | 14665246 | No Genes Found |
| BTA-32346-no-rs | rs798983657 | 13 | 38530393 | DTD1, DZANK1, POLR3F, RBBP9, SEC23B, SMIM26 |
| ARS-BFGL-NGS-113343 | rs478776251 | 13 | 42043229 | CST11, CST8, CSTL1, GZF1, MGC133636, NAPB, NXT1, bta-mir-2285cc |
| BTB-00528306 | rs382827869 | 13 | 53205470 | PDYN, SIRPA |
| ARS-BFGL-NGS-75973 | rs478978144 | 13 | 63769995 | AHCY, ITCH |
| ARS-BFGL-NGS-98521 | rs480706609 | 13 | 66240806 | GHRH, MANBAL, MROH8, RPN2, SRC |
| ARS-BFGL-NGS-112656 | rs1116534779 | 13 | 66336246 | MANBAL, SRC |
| IDBv21300002062 | rs5346309413 | 13 | 66872951 | RPRD1B, TTI1, VSTM2L |
| ARS-BFGL-NGS-116471 | rs110260940 | 13 | 67178078 | BPI, KIAA1755, LBP |
| ARS-BFGL-NGS-3823 | rs5346444400 | 13 | 67530414 | ACTR5, ADIG, PPP1R16B, RALGAPB, SLC32A1 |
| ARS-BFGL-NGS-37880 | rs443047886 | 13 | 75742150 | EYA2, ZMYND8 |
| ARS-BFGL-NGS-23787 | rs5476834406 | 13 | 76042839 | NCOA3 |
| ARS-BFGL-NGS-52851 | rs716440032 | 13 | 77161984 | ARFGEF2, CSE1L, STAU1 |
| ARS-BFGL-NGS-101078 | rs448971855 | 13 | 81297978 | No Genes Found |
| BovineHD1300023674 | rs471165307 | 13 | 81680122 | CYP24A1, PFDN4 |
| IDBv21400002069 | rs3423372646 | 14 | 1802264 | ADGRB1, ARC, JRK, PSCA |
| ARS-BFGL-BAC-10345 | rs478595173 | 14 | 6133529 | No Genes Found |
| ARS-BFGL-BAC-8730 | rs523607510 | 14 | 7926827 | ST3GAL1 |
| ARS-BFGL-NGS-101433 | rs467056142 | 14 | 16224327 | ANXA13, KLHL38 |
| ARS-BFGL-NGS-37911 | rs723093163 | 14 | 16387114 | ATAD2, FBXO32, NTAQ1 |
| ARS-BFGL-NGS-103654 | rs137415187 | 14 | 17109001 | No Genes Found |
| BTA-75794-no-rs | rs132766499 | 14 | 19380690 | H3-5, PRKDC, SPIDR |
| ARS-BFGL-BAC-1302 | rs210286405 | 14 | 27604507 | NKAIN3 |
| BTB-00562922 | rs717383642 | 14 | 30449596 | CRH, DNAJC5B, TRIM55 |
| BTB-00563836 | rs716933831 | 14 | 30818549 | ADHFE1, MYBL1, U6, VCPIP1, VXN |
| UA-IFASA-6858 | rs482353075 | 14 | 45843102 | SAMD12, bta-mir-2489 |
| IDBv31400007695 | rs209916133 | 14 | 46835065 | MED30 |
| BTB-00567299 | rs134018691 | 14 | 46845423 | MED30 |
| BTB-01582460 | rs132911027 | 14 | 52472130 | No Genes Found |
| ARS-BFGL-NGS-98209 | rs447155825 | 14 | 80618301 | No Genes Found |
| BTB-00578376 | rs382553442 | 15 | 5728691 | MMP1, MMP12, MMP13, MMP3 |
| ARS-BFGL-BAC-4818 | rs475324934 | 15 | 10975086 | No Genes Found |
| ARS-BFGL-NGS-114536 | rs714472414 | 15 | 26660750 | No Genes Found |
| BTB-01559194 | rs471619760 | 15 | 36950873 | No Genes Found |
| ARS-BFGL-NGS-101151 | rs439655772 | 15 | 45842102 | OR10A4, OR10A5, OR10A5G, OR2D2, OR2D3, OR6A2, U6, ZNF214, ZNF215 |
| IDBv21500002166 | rs208781829 | 15 | 54219186 | NEU3, OR2AT2C, RNF169, SPCS2, XRRA1 |
| ARS-BFGL-NGS-48903 | rs41771871 | 15 | 56842162 | ANO3 |
| BTB-00608390 | rs722458186 | 15 | 59183752 | No Genes Found |
| BovineHD1500017346 | rs718622655 | 15 | 60356466 | No Genes Found |
| BTB-00613090 | rs436876987 | 15 | 69663711 | No Genes Found |
| ARS-BFGL-NGS-69487 | rs445614610 | 16 | 20704034 | No Genes Found |
| ARS-BFGL-NGS-26656 | rs467789178 | 16 | 21092478 | GPATCH2, SPATA17 |
| ARS-BFGL-NGS-54368 | rs519138899 | 16 | 27572128 | CNIH4, NVL, WDR26 |
| BovineHD1600009289 | rs723174786 | 16 | 32605798 | CATSPERE, DESI2 |
| ARS-BFGL-NGS-97475 | rs109373658 | 16 | 37249521 | F5, SELL, SELP |
| ARS-BFGL-NGS-40844 | rs482861626 | 16 | 45229504 | No Genes Found |
| BTA-39051-no-rs | rs518866806 | 16 | 47405443 | NPHP4 |
| BTB-00643802 | rs133828136 | 16 | 48725717 | AJAP1 |
| BovineHD1600014225 | rs452526355 | 16 | 51118732 | ACAP3, ANKRD65, ATAD3A, AURKAIP1, CCNL2, CPTP, DVL1, FNDC10, INTS11, MIB2, MRPL20, MXRA8, PUSL1, SSU72, TAS1R3, TMEM240, TMEM88B, VWA1 |
| ARS-BFGL-NGS-93273 | rs458007442 | 16 | 51299881 | ACAP3, B3GALT6, C16H1orf159, C1QTNF12, CPTP, INTS11, PUSL1, SCNN1D, SDF4, TNFRSF18, TNFRSF4, TTLL10, UBE2J2 |
| ARS-BFGL-NGS-22730 | rs715686757 | 16 | 53922176 | PDPN, PRDM2 |
| BovineHD1600021593 | rs467528557 | 16 | 75388268 | CD34, PLXNA2 |
| ARS-BFGL-NGS-102714 | rs442519505 | 16 | 77058280 | NEK7 |
| BTA-06911-no-rs | rs482446646 | 16 | 79063017 | ZNF281 |
| ARS-BFGL-NGS-36059 | rs207513014 | 16 | 79896422 | ADIPOR1, CYB5R1, KDM5B, KLHL12, MGAT4E, MGAT4F, PKP1, RABIF |
| ARS-BFGL-NGS-22126 | rs515750828 | 17 | 10498926 | ARHGAP10, EDNRA, PRMT9, TMEM184C |
| ARS-BFGL-NGS-29563 | rs435493948 | 17 | 18574363 | ELF2, NOCT |
| ARS-BFGL-NGS-66923 | rs384404549 | 17 | 18910914 | No Genes Found |
| ARS-BFGL-NGS-119157 | rs470321430 | 17 | 20275502 | PCDH18 |
| ARS-BFGL-NGS-108496 | rs386126467 | 17 | 25441346 | No Genes Found |
| ARS-BFGL-NGS-115427 | rs798961923 | 17 | 46088305 | ADGRD1 |
| ARS-BFGL-NGS-102781 | rs207744546 | 17 | 48084367 | GLT1D1, TMEM132D |
| ARS-BFGL-NGS-14183 | rs714709163 | 17 | 54456978 | FAM216A, GPN3, HVCN1, PPTC7, RAD9B, TCTN1, VPS29 |
| ARS-BFGL-NGS-11333 | rs466959422 | 17 | 62662034 | COX6A1, GATC, MSI1, PLA2G1B, PXN, SIRT4, SRSF9, TRIAP1, U4 |
| ARS-BFGL-NGS-23799 | rs208277791 | 17 | 64754159 | WSCD2, bta-mir-6521 |
| BovineHD1700021251 | rs435720289 | 17 | 72742097 | C17H22orf39, CDC45, CLDN5, GNB1L, GP1BB, HIRA, MRPL40, SEPTIN5, TBX1, UFD1 |
| ARS-BFGL-NGS-113725 | rs523529725 | 18 | 2685718 | BCAR1, BCNT2, CFDP1, CFDP2 |
| ARS-BFGL-NGS-64834 | rs5477048437 | 18 | 10289114 | MLYCD, NECAB2, OSGIN1, SLC38A8 |
| ARS-BFGL-NGS-53926 | rs723880977 | 18 | 10642175 | ATP2C2, WFDC1 |
| ARS-BFGL-NGS-17185 | rs433425346 | 18 | 22379853 | FTO |
| ARS-BFGL-NGS-23701 | rs446102036 | 18 | 22599071 | IRX3 |
| ARS-BFGL-NGS-38367 | rs516729702 | 18 | 23916486 | CAPNS2, CES1, LPCAT2, SLC6A2, U6 |
| BTA-42979-no-rs | rs467893703 | 18 | 32517821 | No Genes Found |
| ARS-BFGL-NGS-23924 | rs5520888543 | 18 | 39549809 | CHST4, TAT, TLE7, U6, ZNF19, ZNF23 |
| BTB-00712788 | rs1115861277 | 18 | 40116972 | No Genes Found |
| ARS-BFGL-NGS-57914 | rs211171260 | 18 | 43726997 | CEBPA, CEBPG, LRP3, PEPD, SLC7A10 |
| ARS-BFGL-BAC-35025 | rs797208701 | 18 | 48038041 | C18H19orf33, DPF1, KCNK6, PPP1R14A, SIPA1L3, SPINT2, U6, YIF1B |
| ARS-BFGL-BAC-28016 | rs5352406578 | 18 | 52003816 | IRGC, KCNN4, LYPD5, SMG9, ZNF283, ZNF404 |
| ARS-BFGL-NGS-39575 | rs382815944 | 18 | 56775122 | ACP4, C18H19orf48, C18H19orf81, CLEC11A, LRRC4B, SHANK1, SMIM47, SYT3 |
| BovineHD1800018004 | rs380848834 | 18 | 62412976 | EPS8L1, GP6, NCR1, RDH13 |
| ARS-BFGL-NGS-15837 | rs5352982913 | 18 | 62591127 | FCAR, KIR2DL5A, KIR3DL2, NCR1 |
| BovineHD1800019044 | rs385562301 | 18 | 65384131 | U6, ZNF814 |
| ARS-BFGL-NGS-102974 | rs517732944 | 19 | 1822133 | No Genes Found |
| UA-IFASA-7543 | rs378523198 | 19 | 5057128 | COX11, STXBP4, TOM1L1 |
| IDBv31900008418 | rs1114490662 | 19 | 20964677 | ANKRD13B, CORO6, SSH2, U6 |
| IDBv31900008428 | rs210417230 | 19 | 20977359 | SSH2, U6 |
| ARS-BFGL-NGS-103531 | rs381571778 | 19 | 21006570 | SSH2, U6 |
| UA-IFASA-8469 | rs434466828 | 19 | 24245251 | ASPA, CTNS, OR1E2, SHPK, SPATA22, TRPV1, TRPV3 |
| ARS-BFGL-NGS-38067 | rs5381771326 | 19 | 27444684 | ATP1B2, DNAH2, EFNB3, KDM6B, SAT2, SHBG, TP53, WRAP53 |
| ARS-BFGL-NGS-114201 | rs459173199 | 19 | 27576066 | CHD3, CNTROB, CYB5D1, DNAH2, GUCY2D, KCNAB3, KDM6B, NAA38, RNF227, TMEM88, TRAPPC1 |
| ARS-BFGL-NGS-101671 | rs465018768 | 19 | 28366979 | CCDC42, MFSD6L, NTN1, PIK3R5, PIK3R6 |
| IDBv21900002499 | rs468357646 | 19 | 43045807 | AARSD1, BRCA1, G6PC1, IFI35, NBR1, PTGES3L, RND2, RPL27, RUNDC1, VAT1 |
| IDBv21900002505 | rs716454574 | 19 | 43046856 | AARSD1, BRCA1, G6PC1, IFI35, NBR1, PTGES3L, RND2, RPL27, RUNDC1, VAT1 |
| ARS-BFGL-NGS-20682 | rs524966240 | 19 | 43904899 | ASB16, G6PC3, HDAC5, HROB, LSM12, NAGS, PPY, PYY, PYY2, TMEM101 |
| IDBv21900003050 | rs136653984 | 19 | 48967145 | BPTF, C19H17orf58, KPNA2, SMURF2 |
| ARS-BFGL-BAC-33563 | rs797988958 | 19 | 57017362 | BTBD17, CD300A, DNAI2, GPR142, GPRC5C, KIF19 |
| BovineHD1900017514 | rs383125604 | 19 | 61022958 | MAP2K6 |
| IDBv21900003117 | rs445248962 | 19 | 61707432 | ARSG, FAM20A, MGC134105, PRKAR1A, WIPI1 |
| BTA-86837-no-rs | rs380029808 | 20 | 16044811 | No Genes Found |
| ARS-BFGL-NGS-71000 | rs209765524 | 20 | 17742195 | U6 |
| BTB-01524822 | rs797868095 | 20 | 18719808 | DEPDC1B |
| ARS-BFGL-NGS-4476 | rs135615896 | 20 | 18950015 | PDE4D |
| BTB-01501284 | rs716313994 | 20 | 19262754 | PDE4D |
| ARS-BFGL-NGS-18991 | rs210655927 | 20 | 19710624 | PDE4D |
| BovineHD2000006635 | rs469717799 | 20 | 22158307 | GPBP1 |
| ARS-BFGL-NGS-30073 | rs525134984 | 20 | 24697526 | ARL15, HSPB3 |
| ARS-BFGL-NGS-110286 | rs110629176 | 20 | 25689084 | No Genes Found |
| ARS-BFGL-NGS-116034 | rs715510568 | 20 | 50885247 | No Genes Found |
| ARS-BFGL-NGS-26451 | rs209679462 | 20 | 64271255 | SEMA5A, U3 |
| BTA-20615-no-rs | rs719526899 | 21 | 9741507 | No Genes Found |
| BovineHD2100002321 | rs462652646 | 21 | 9742241 | No Genes Found |
| BovineHD2100004177 | rs446207851 | 21 | 15418156 | No Genes Found |
| ARS-BFGL-NGS-103603 | rs110081649 | 21 | 19312826 | NTRK3 |
| ARS-BFGL-NGS-84831 | rs380974070 | 21 | 21831416 | BLM, FES, FURIN, HDDC3, MAN2A2 |
| ARS-BFGL-NGS-12511 | rs379748343 | 21 | 33601152 | C21H15orf39, PPCDC, SCAMP5 |
| BTB-00822762 | rs450388100 | 21 | 49165337 | GEMIN2, MIA2, PNN, SEC23A, TRAPPC6B |
| ARS-BFGL-NGS-67889 | rs517890836 | 21 | 52239094 | No Genes Found |
| BovineHD2100016524 | rs517991811 | 21 | 57516455 | CHGA, GOLGA5, ITPK1 |
| BovineHD2100017392 | rs517710317 | 21 | 59979582 | CLMN, DICER1 |
| ARS-BFGL-NGS-67767 | - | 21 | 70272221 | No Genes Found |
| BTA-55263-no-rs | rs5349561800 | 22 | 3126680 | RBMS3 |
| ARS-BFGL-NGS-77874 | rs1114928489 | 22 | 8223349 | U6 |
| ARS-BFGL-BAC-30382 | rs435820978 | 22 | 21495296 | BHLHE40, ITPR1 |
| BTA-109833-no-rs | rs797478346 | 22 | 37730087 | C22H3orf49, SNTN, SYNPR |
| ARS-BFGL-BAC-35143 | rs520548248 | 22 | 41480732 | FHIT |
| ARS-BFGL-NGS-19029 | rs524731644 | 22 | 49987199 | CACNA2D2, CYB561D2, HYAL1, HYAL2, HYAL3, IFRD2, NPRL2, RASSF1, SEMA3B, TMEM115, TUSC2, ZMYND10 |
| UA-IFASA-5612 | rs717175806 | 22 | 53944830 | LARS2, TMEM158 |
| ARS-BFGL-NGS-119941 | rs1114555822 | 22 | 60543119 | ALDH1L1, C22H3orf22, CHST13, KLF15, SLC41A3, UROC1, ZXDC |
| ARS-BFGL-NGS-105794 | - | 22 | 61199665 | No Genes Found |
| ARS-BFGL-NGS-59707 | rs208647588 | 23 | 2570211 | PRIM2, U6 |
| ARS-BFGL-NGS-38075 | rs456911878 | 23 | 3360714 | bta-mir-2375 |
| ARS-BFGL-NGS-43411 | rs474871253 | 23 | 3885583 | No Genes Found |
| ARS-BFGL-NGS-119695 | rs211036946 | 23 | 4170083 | COL21A1 |
| ARS-BFGL-NGS-111195 | rs3423513824 | 23 | 15375447 | FOXP4, MDFI |
| BovineHD2300006947 | rs522448238 | 23 | 25089544 | GSTA1, GSTA2, GSTA3, GSTA5, TMEM14A |
| ARS-BFGL-NGS-97747 | rs481232670 | 23 | 27939837 | C23H6orf15, CCHCR1, CDSN, MIC1, MIC2, POU5F1, PSORS1C2, TCF19 |
| BovineHD2300008530 | rs208996363 | 23 | 29874313 | OR10AL40, OR10AL43, OR2AD1, OR2AD1B, OR2J3B, OR2W1 |
| BovineHD4100016195 | rs134882908 | 23 | 39413573 | DEK, KDM1B, NHLRC1, RNF144B, TPMT, U6 |
| ARS-BFGL-NGS-4837 | rs720804251 | 23 | 41901234 | No Genes Found |
| BTB-00867439 | rs378897399 | 23 | 45945169 | No Genes Found |
| BovineHD2400003409 | rs452570273 | 24 | 12158958 | No Genes Found |
| ARS-BFGL-NGS-85237 | rs210504399 | 24 | 15489455 | No Genes Found |
| BTB-00882676 | rs472910638 | 24 | 17144207 | No Genes Found |
| ARS-BFGL-NGS-46034 | rs474554675 | 24 | 20787578 | FHOD3, MOCOS, U6 |
| ARS-BFGL-NGS-56400 | rs3423526558 | 24 | 20973161 | ELP2, MOCOS, RPRD1A, SLC39A6 |
| ARS-BFGL-NGS-102332 | rs467863976 | 24 | 21918990 | MAPRE2 |
| ARS-BFGL-BAC-28639 | rs1114526236 | 24 | 23774345 | CCDC178 |
| ARS-BFGL-NGS-115494 | - | 24 | 30503642 | KCTD1, TAF4B |
| ARS-BFGL-NGS-41583 | rs434901959 | 24 | 34485175 | ABHD3, MIB1, bta-mir-133a-2 |
| BTB-00888688 | rs385648774 | 24 | 37313598 | LPIN2, MYL12A, MYOM1 |
| BTB-01189219 | rs208457191 | 24 | 37459437 | MYL12A, MYL12B, MYOM1, TGIF1 |
| ARS-BFGL-BAC-44783 | rs455230726 | 24 | 40983360 | PTPRM, RAB12, bta-mir-2285o-4 |
| ARS-BFGL-NGS-101197 | rs458580987 | 24 | 41358011 | ANKRD12, NDUFV2 |
| ARS-BFGL-NGS-29353 | rs438067587 | 24 | 42236118 | APCDD1, NAPG, PIEZO2 |
| ARS-BFGL-NGS-112695 | rs452064878 | 24 | 48618537 | CTIF, SMAD7 |
| ARS-BFGL-NGS-73260 | rs454798118 | 24 | 59177468 | MC4R |
| BovineHD2500000155 | rs207815174 | 25 | 709580 | ANTKMT, CCDC78, CHTF18, CIAO3, GNG13, HAGHL, LMF1, METRN, MSLN, RPUSD1, SOX8 |
| ARS-BFGL-NGS-117215 | rs133399410 | 25 | 22890173 | AQP8, ZKSCAN2 |
| ARS-BFGL-NGS-110948 | rs437607163 | 25 | 26747553 | FBRS, ITGAL, PRR14, SNORA30, SRCAP, ZNF688, ZNF689, ZNF768 |
| ARS-BFGL-NGS-53976 | rs457386240 | 25 | 29485851 | GALNT17 |
| BTA-60358-no-rs | rs442220457 | 25 | 39197736 | AP5Z1, FOXK1, MMD2, PAPOLB, RADIL, bta-mir-2890 |
| ARS-BFGL-NGS-73431 | rs438673481 | 26 | 2648036 | No Genes Found |
| ARS-BFGL-NGS-47701 | rs42084163 | 26 | 7251671 | PRKG1 |
| BTB-01783203 | rs524364315 | 26 | 9248661 | MINPP1, PAPSS2 |
| IDBv32600009708 | rs876455042 | 26 | 10478102 | ANKRD22, LIPK, LIPM, LIPN, STAMBPL1 |
| ARS-BFGL-NGS-114539 | rs482354814 | 26 | 18808408 | ANKRD2, AVPI1, C10orf62, HOGA1, MARVELD1, MMS19, MORN4, PI4K2A, UBTD1, ZFYVE27 |
| ARS-BFGL-NGS-115980 | rs42516381 | 26 | 20065602 | HPSE2 |
| ARS-BFGL-NGS-10307 | rs211430895 | 26 | 20259486 | CNNM1, HPSE2 |
| ARS-BFGL-NGS-12828 | rs525785186 | 26 | 37018466 | HSPA12A, PNLIP, PNLIPRP1, PNLIPRP2, SPMIP5, bta-mir-2395 |
| ARS-BFGL-NGS-10498 | rs384797407 | 26 | 41341146 | No Genes Found |
| ARS-BFGL-NGS-32861 | rs133531927 | 26 | 43957191 | OAT |
| ARS-BFGL-NGS-117809 | rs717170811 | 26 | 43981400 | OAT |
| ARS-BFGL-NGS-53327 | rs452590576 | 26 | 45825261 | ADAM12 |
| BovineHD2600014616 | rs5358016181 | 26 | 50459088 | TCERG1L |
| ARS-BFGL-NGS-35140 | rs5407379770 | 27 | 2748297 | CSMD1, U6 |
| BTB-01314387 | rs446358942 | 27 | 7450898 | GPM6A |
| BovineHD2700003073 | rs432115073 | 27 | 10304257 | SNORD22 |
| ARS-BFGL-NGS-23308 | rs451629187 | 27 | 14526116 | STOX2 |
| IDBv22700003800 | rs798764297 | 27 | 14543328 | STOX2 |
| IDBv22700003811 | rs518021379 | 27 | 14551869 | STOX2 |
| ARS-BFGL-NGS-102850 | rs134675634 | 27 | 14931898 | IRF2 |
| UA-IFASA-1830 | rs722179292 | 27 | 17871967 | No Genes Found |
| BTB-02036330 | rs518324159 | 27 | 21577683 | TUSC3 |
| BTB-00961082 | rs136460596 | 27 | 21615584 | TUSC3 |
| BTB-00960677 | rs435614758 | 27 | 21750558 | SGCZ |
| ARS-BFGL-NGS-117840 | rs453946413 | 27 | 41418572 | THRB |
| ARS-BFGL-NGS-111221 | rs797384987 | 28 | 25020359 | CCAR1, STOX1, TET1, U6 |
| BovineHD2800009889 | rs133563697 | 28 | 36215397 | U2 |
| ARS-BFGL-NGS-118113 | rs801408338 | 28 | 39482527 | CCSER2, U6 |
| BTB-00991289 | rs466581916 | 28 | 41498417 | ADIRF, BMPR1A, MMRN2, SNCG |
| ARS-BFGL-NGS-56314 | rs43332062 | 29 | 4944723 | No Genes Found |
| ARS-BFGL-NGS-35685 | rs521250570 | 29 | 6689993 | GRM5 |
| BTA-03099-rs29010866 | rs521001118 | 29 | 9595450 | CCDC83, PICALM |
| ARS-BFGL-NGS-31939 | rs482638940 | 29 | 14153957 | No Genes Found |
| BTB-00426200 | rs441125485 | 29 | 14664124 | No Genes Found |
| ARS-BFGL-NGS-73946 | rs5358515758 | 29 | 14828179 | No Genes Found |
| ARS-BFGL-NGS-79435 | rs432861271 | 29 | 16216928 | No Genes Found |
| ARS-BFGL-NGS-57426 | rs456016340 | 29 | 23509444 | NELL1 |
| ARS-BFGL-NGS-106691 | rs1114172936 | 29 | 29952558 | KIRREL3 |
| BTA-65537-no-rs | rs461330841 | 29 | 33407833 | OPCML |
| BovineHD2900010417 | rs464711658 | 29 | 34737487 | NTM |
| IDBv22900003972 | rs384520045 | 29 | 50047035 | BRSK2, DUSP8, MOB2 |
| ARS-BFGL-NGS-40378 | rs439763128 | 29 | 50296573 | MUC2 |
| BovineHD2900014811 | rs715902931 | 29 | 50783100 | ANO9, CDHR5, HRAS, IRF7, LMNTD2, LRRC56, PHRF1, PKP3, PTDSS2, RASSF7, RNH1, SCT, SIGIRR, bta-mir-210 |
| BovineHD2900014833 | rs435770006 | 29 | 50854859 | ANO9, B4GALNT4, HRAS, LRRC56, PKP3, PTDSS2, RNH1, SIGIRR |

^1^The table lists significant Single Nucleotide Polymorphisms (SNP) identified in the study. For each SNP, the table provides the unique identifier (SNP ID and rsID), chromosomal location (Chromosome and Position), and associated genes (Total: 947 unique genes).

Fig. S1: Feature importance of top-ranked SNP ordered by their mean absolute SHapley Additive exPlanations (SHAP) values derived from the Random Forest model. Blue bars indicate markers associated with a decrease in the Estimated Breeding Value (EBV) for Residual Feed Intake (positive effect), while pink bars indicate markers associated with an increase in EBV (negative effect).

Fig. S2: STRING interaction network for genes linked to SNP associated using Random Forest to Residual Feed Intake in Dairy cattle with the highest degree of centrality represented by node *FTSJ3*.

Fig. S3: Feature importance of top-ranked SNP ordered by their mean absolute SHapley Additive exPlanations (SHAP) values derived from the Multi-Layer Perceptron model. Blue bars indicate markers associated with a decrease in the Estimated Breeding Value (EBV) for Residual Feed Intake (positive effect), while pink bars indicate markers associated with an increase in EBV (negative effect).

Fig S4: STRING interaction network for genes linked to SNP associated using Multi-Layer Perceptron to Residual Feed Intake in Dairy cattle with the highest degree of centrality represented by node *TP53*.
